# Limit Allogeneic Blood Use with Routine Re-use of Patient's Own Blood: A Prospective, Randomized, Controlled Trial in Total Hip Surgery

**DOI:** 10.1371/journal.pone.0044503

**Published:** 2012-09-13

**Authors:** Bregje J. W. Thomassen, Peter Pilot, Vanessa A. B. Scholtes, Josef G. Grohs, Ketil Holen, Elvira Bisbe, Rudolf W. Poolman

**Affiliations:** 1 Department of Orthopaedic Surgery, Medical Center Haaglanden, The Hague, The Netherlands; 2 Department of Orthopaedic Surgery, Reinier de Graaf Hospital, Delft, The Netherlands; 3 Department of Orthopaedic Surgery, Onze Lieve Vrouwe Gasthuis, Amsterdam, The Netherlands; 4 Department of Orthopaedic Surgery, Medical University Vienna, Vienna, Austria; 5 Department of Orthopaedic Surgery, St. Olavs Hospital, Trondheim, Norway; 6 Department of Anesthesiology, Hospital Universitario Mar-Esperança, Barcelona, Spain; University of Ottawa, Canada

## Abstract

**Background:**

There are risks related to blood incompatibility and blood-borne diseases when using allogeneic blood transfusion. Several alternatives exist today, one of which, used for autologous blood salvage perioperatively, is the Sangvia Blood Management System. This study was designed to investigate the efficacy of the system and to add data to previously reported safety results.

**Methodology/Principal Findings:**

Two hundred sixteen patients undergoing primary or revision total hip arthroplasty (THA) were enrolled in this randomized, controlled, assessor-blinded multicenter study. Randomization was either autologous blood transfusion (Sangvia group) or no use of autologous blood (Control group), both in combination with a transfusion protocol for allogeneic transfusion. Patients were followed during hospital stay and at two months after discharge. The primary outcome was allogeneic blood transfusion frequency. Data on blood loss, postoperative hemoglobin/hematocrit, safety and quality of life were also collected. The effectiveness analysis including all patients showed an allogeneic blood transfusion rate of 14% in both groups. The efficacy analysis included 197 patients and showed a transfusion rate of 9% in the Sangvia group as compared to 13% in the Control group (95%CI −0.05–0.12, p = 0.5016). A mean of 522 mL autologous blood was returned in the Sangvia group and lower calculated blood loss was seen. 1095 mL vs 1285 mL in the Control group (95%CI 31–346, p = 0.0175). No differences in postoperative hemoglobin was detected but a lower hematocrit reduction after surgery was seen among patients receiving autologous blood. No relevant differences were found for safety parameters or quality of life.

**Conclusions/Significance:**

General low use of allogeneic blood in THA is seen in the current study of the Sangvia system used together with a transfusion protocol. The trial setting is under-powered due to premature termination and therefore not able to verify efficacy for the system itself but contributes with descriptive data on safety.

**Trial Registration:**

Clinicaltrials.gov NCT00822588

## Introduction

In major orthopedic procedures, there is considerable blood loss during and after surgery which causes acute postoperative anemia and often leads to a rising need for allogeneic blood transfusions. Allogeneic blood transfusion is not a risk-free therapy, as it is associated with potential risks of matching errors, down-modulation of the immune system [1], [2], increased infection rate [3], [4], absence of clotting factors and transmission of infectious diseases [5], [6], which may result in poorer postoperative outcomes and higher mortality [7], [8]. In addition, some patients refuse to have an allogeneic blood transfusion for religious reasons and allogeneic blood is a limited and increasingly expensive resource [9]. To minimize these disadvantages, a variety of alternative interventions has been developed to reduce the need for allogeneic transfusions. These interventions are generally either agents to diminish blood loss (e.g. tranexamic acid) [10], [11], agents that promote red blood cell production (e.g. erythropoietin) [12] or techniques for re-infusing the patient's own blood (e.g. cell salvage) [13]–[16]. A systematic review of previously studied cell salvage systems suggested that their use is efficacious in reducing the need for allogeneic transfusion in cardiac and orthopaedic surgery even though it concluded that the methodology was poor [17].

Both ‘filtered’ and ‘washed’ cell salvage systems are commonly used and known contraindications are mainly related to the quality of the collected blood. While it is known that salvaged blood is laden with complement split products, interleukins, various inflammatory mediators and fat particles [18]–[21] the clinical implication of these factors is not clear.

This study was designed to evaluate the clinical efficacy of a new device for cell salvage, i.e. the Sangvia™ Blood Management System (Astra Tech AB, Mölndal, Sweden), by using a scientifically sound research methodology. The study was also done to further investigate the safety aspect of ‘filtered’ cell salvage and to confirm previously reported safety findings for this new device for cell salvage [20], [21].

## Methods

This was an international multicenter, prospective, assessor-blinded, randomized, controlled trial with an adaptive statistical study design. Six European hospitals were involved, located in The Netherlands (three clinics), Spain, Norway and Austria. The protocol for this trial and supporting CONSORT checklist are available as supporting information; see CONSORT Checklist S1 and Protocol S1.

### Participants

For inclusion in the study, patients had to be scheduled for primary or revision total hip arthroplasty and be classified as American Society of Anesthesiology (ASA) class I, II or III. The following criteria excluded the patients from participation in the study:

Exclusion due to ethical concern included previous randomization in this study, involvement in the planning and/or conduct of this study, and participation in an interfering study.
Exclusion due to safety concerns included current symptoms of hemophilia and contraindications for autologous blood use, i.e. hyperkalaemia, current systemic infection or local infection in the operation field or impaired renal function (elevated creatinine/clearance levels), known malignancy in the last five years and expected use of cytotoxic drugs.
Exclusion due to expected impact on outcome included untreated anemia (hemoglobin (Hb) level <11 g/dL), revision total hip arthroplasties with expected serious bone grafting, and use of other alternatives for blood conservation such as recombinant erythropoietin, fibrin sealant, aprotinin and other autologous blood transfusion.

Use of tranexamic acid was allowed if routinely used in the individual clinic and thus equally distributed between the treatment groups. The decision for tranexamic acid use had to be made before randomization.

### Ethics

Written informed consent was obtained from all participating patients. The study was approved by applicable local ethics committees before its initiation and was conducted in accordance with the Declaration of Helsinki, ICH/Good Clinical Practice, and regulatory requirements. The following ethics committees approved the study: Medisch-ethische commissie at Onze lieve vrouwe gasthuis (reference WO 09.033), METC Zuidwest Holland (reference 09–031), Medisch Centrum Haaglanden (reference RVB/RZ/1444), Reinier de Graaf Groep (reference CZ/CS/2009-086), CEIC-IMAS (reference YA-DRA-0001, version 2.0, date 12/01/2009), Det medisinske fakultet Regional komite for medisinsk og helsefaglig forskningsetikk Helseregion Midt-Norge (reference 4.2009.421), Ethik-kommission der Medizinischen Universität Wien und des Allgemeinen Krankenhauses Der stadt Wien AKH (reference 011/2009).

### Interventions

Prior surgery patients scheduled for primary or revision total hip arthroplasty (THA) were randomized to receive either autologous blood transfusion (Sangvia group) or no use of autologous blood (Control group) in combination with using a transfusion protocol limiting allogeneic blood transfusions to patients with hemoglobin (Hb) values below 8.5 g/dL or significant clinical symptoms of anemia. Surgery was performed by orthopedic surgeons following their routine procedures. The Sangvia group used the Sangvia™ Blood Management System (Astra Tech AB, Mölndal, Sweden) for surgery. The system was used according to the manufacturer's instructions for both intra-operative and postoperative autologous blood collection and transfusion. Details about the system are published in Kvarström et al. and Stachura et al. [20], [21]. Postoperative drains were used in both groups to standardize postoperative routines and minimize differences between the two treatment groups, i.e. the Sangvia drain for postoperative autologous blood collection and transfusion in the Sangvia group and a regular postoperative low-vacuum drain (Bellovac™, Astra Tech AB, Mölndal, Sweden) in the Control group. Both drainage systems were used until the first postoperative morning. Before patient recruitment started ten systems were used to train operating room staff at each of the study sites.

Rehabilitation followed the standard procedure at each specific hospital.

### Outcomes

The primary outcome measure was allogeneic blood transfusion frequency, given as a relative percentage, and measured at the day of discharge. Allogeneic blood transfusions were also described by the number of transfusion decisions taken for a patient, the transfusion volume and the calculated transfusion index (total number of units per transfused patient).

Secondary outcome measures included blood loss, postoperative Hb and hematocrit (Hct), safety and quality of life. The intra-operative blood loss was estimated by the surgeon by evaluating intra-operative cell saving volumes, waste suction volumes and weighing gauzes. The research assistant estimated blood loss after surgery based on drain volumes. The sum of intra- and postoperative blood loss represents the total value of estimated blood loss. In addition to the estimated values, blood loss was calculated on the basis of blood volume and Hct values [22], [23], i.e. calculated blood loss (mL) = [Total blood volume (mL)×(Hct_pre-op_ – Hct_post-op_)]/[(Hct_pre-op_+Hct_post-op_)/2]. Total blood volume was calculated in liters by the formula (0.3669×height (m^3^))+(0.03219×weight (kg))+0.6041 for men and (0.3561×height (m^3^))+(0.03308×weight (kg))+0.1833 for women.

Safety data included vital signs (heart rate, blood pressure, temperature), laboratory variables (potassium, sodium, creatinine and Glomerular Filtration Rate) and adverse events classified by severity and causality. Quality of life was assessed by the EuroQol (EQ-5D) health status questionnaire [24], [25]. All patients were followed during surgery and their hospital stay with outcome measures collected pre-operatively, at three hours, on one, two and four days after surgery, and on the day of discharge. A final check-up to fill out the EQ-5D questionnaire was done two months after surgery.

### Sample size

The study used an adaptive statistical design where a preplanned interim analysis on half of the population was done to confirm or reject sample size estimations. The null hypothesis tested for rejection was if the allogeneic transfusion frequency was equal in the Sangvia and in the Control group. The initial sample size calculation (power 90%, 5% two-sided level of significance) was based on the literature and normal use of allogeneic blood in the Control group was estimated to be 21% [13], [26]–[28]. The expected value for transfusion frequency in the Sangvia group was estimated to be around 7% based on an expected added value to previous reported findings from postoperative autologous systems [13]. The sample size necessary for detection of the expected difference in transfusion frequency was calculated to be 260 patients [29]. A further 40 patients were planned to be included to adjust for non-evaluable patients or drop-outs.

### Randomization

Eligible patients were consecutively randomized to receive either autologous blood transfusion, by the Sangvia™ Blood Management System (Sangvia group), or to no use of autologous blood (Control group).

Treatment allocation was stratified by hospital and type of surgery, i.e. primary or revision arthroplasty. For randomization a block size of 4 and allocation distribution 1∶1 were used. For each hospital, a separate randomization list was generated by a computer and implemented in a web-based login system. The randomization plan and generated list were only known to study personnel not involved in clinical procedures.

The principal investigator/study coordinator randomized the patients as close as possible prior to surgery in the web-based login system. In the majority of cases this was an investigator not involved in surgery. Each patient's actual randomization was checked against the randomization list, inclusion date, surgery date and demographical data to ensure correct implementation and strict consecutive allocation.

### Blinding

To mitigate the risk of bias, the decision for allogeneic blood transfusion was taken on the basis of a transfusion trigger, a Hb value ≤8.5 g/dL, by an assessor unaware of the treatment group. The majority of the clinics used a representative from the blood bank for the decision. In acute situations, i.e. during surgery, the decision had to be taken by the surgeon/anesthesiologist who was aware of the treatment allocation. For all transfusions, indication was registered and for allogeneic blood transfusions with Hb values above 8.5 g/dL, the requirement was for the patient to have clinical symptoms, i.e. signs of anemia, such as tachycardia and/or hypotension. Secondary outcome measures were collected prospectively and analyzed as applicable by independent laboratory personnel unaware of the treatment allocation. Clinical variables such as vital signs, evaluated by personnel in contact with the patient, could not be blinded during the first postoperative day due to the differences in drains used in the Sangvia and Control groups.

### Statistical methods

Analyses were made on both an Intention To Treat (ITT) and a Per Protocol (PP) principle. Demographic and baseline data were based on the results from the ITT procedure. Conclusions related to effectiveness were explored by using the ITT analysis set, and those related to efficacy were based on the results in the PP analysis set. Conclusions related to safety and quality of life were also based on the ITT analysis set. The risk for variability due to the multicenter study design, with for example differences in routines for hospital stay and discharge, was mitigated by use of stratification. Thus the results of the statistical analyses do not present data for each individual clinic.

The Fisher Exact test was used to test frequencies of dichotomous response variables. The non-parametric Wilcoxon Rank Sum rank test was used to analyze differences in continuous response variables between the treatment groups. P-values≤0.05 were considered statistically significant. In addition, 95% confidence intervals (CI) were calculated based on independent sample t-test (equal variances assumed) and presented for comparative data. No correction for multiplicity was made since hypotheses were considered statistically independent.

Tables with descriptive data were generated and hypotheses tested using statistical software PASW Statistics version 18.0 (IBM® SPSS® Statistics).

## Results

### Participant flow

**Figure 1:**
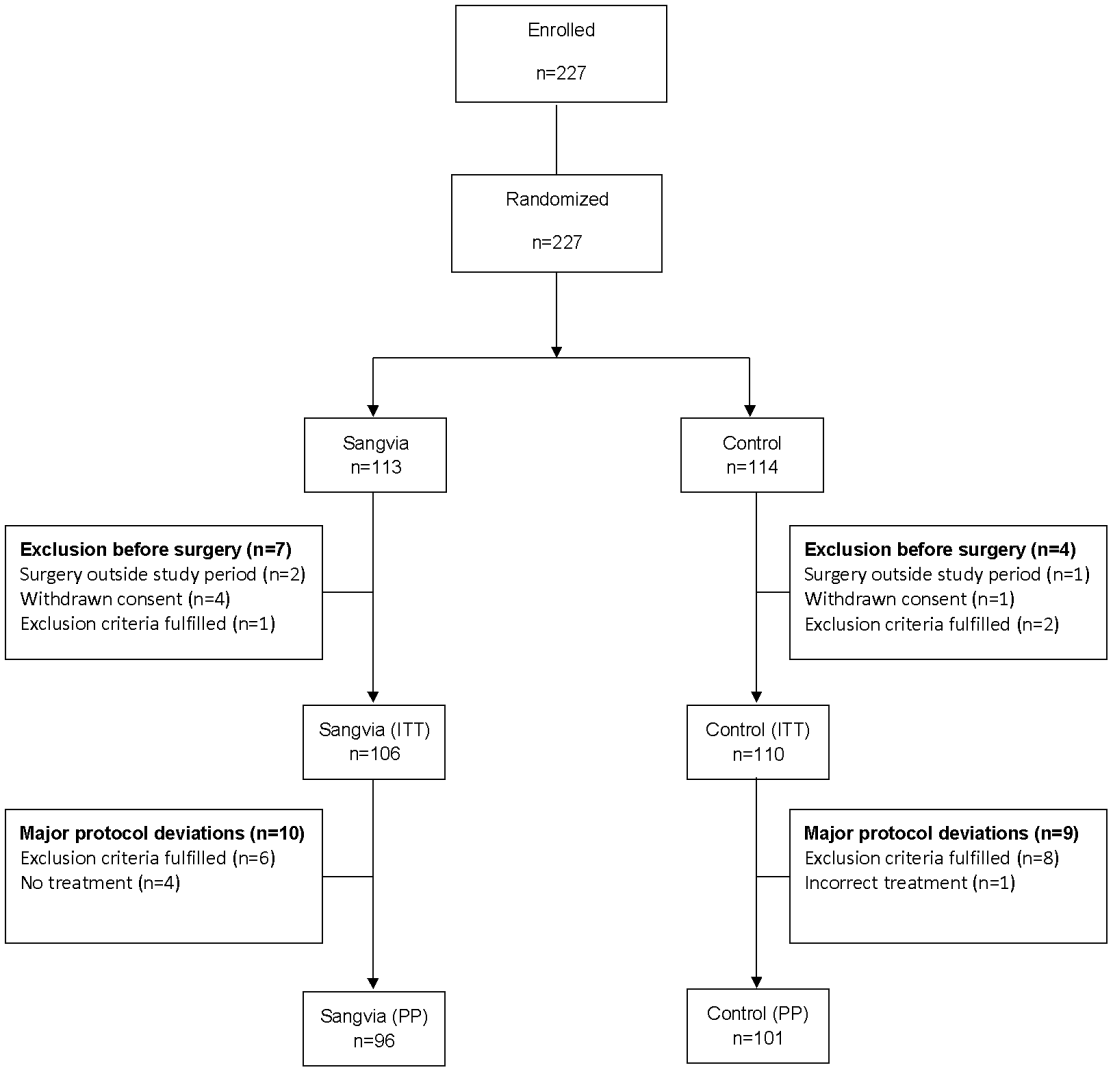
Study patient flow and definition of analysis sets.

The pre-planned interim analysis was performed in 135 patients, 66 in the Sangvia group and 69 in the Control group, and concluded that the transfusion rate in the Control group was 12% instead of the expected 21%. Accordingly, the study was at risk of being under-powered and inconclusive and was prematurely stopped. This resulted in 227 enrolled patients instead of the planned 300. Randomization was done before surgery, and thus all enrolled patients were randomized. Some of the exclusion criteria could only be completely verified after randomization just before, during or after surgery, e.g. local infection in the operation field. Only limited demographical and no follow up data were collected for patients for whom exclusion criteria were identified after randomization but before surgery. Of the 227 patients, this was applicable in 11 patients who were excluded due to withdrawn consent (five patients), exclusion criteria fulfilled (two patients with an ongoing local infection, one patient with a missing creatinine value) and three patients whose surgery was rescheduled after the study was prematurely discontinued. These patients were registered and are presented in Figure 1 but are not represented in any of the analyses due to lacking data. Thus, treatment was allocated and data were collected from a total of 216 patients (ITT analysis), 106 in the Sangvia group and 110 in the Control group.

Major protocol deviations were detected in some patients after treatment, and for that reason two analysis sets were identified, i.e. ITT and PP (Figure 1). Although it is recognized that the ITT analysis set should include all patients intended for treatment the actual ITT analysis set in this study was limited to all treated patients due to missing follow-up data for patients excluded before treatment allocation. The PP analysis set excluded 19 patients for whom major protocol deviations were detected. Ten patients were excluded from the PP analysis in the Sangvia group; other autologous blood devices were used by mistake in four cases, erythropoietin was given by mistake in one case, a preoperatively creatinine level outside the normal range was detected late for one case and no treatment was given due to technical and management difficulties in four cases. Nine patients were excluded from the PP analysis in the Control group; other autologous blood was given by mistake in five cases, preoperatively creatinine level outside normal range was detected late for one case, a preoperatively Hb level below the exclusion criteria was detected late for one case, a history of malignancy was detected late for one case and incorrect treatment allocation was used by mistake in one case, i.e. Sangvia was used. The PP analysis consisted of 197 patients, 96 in the Sangvia group and 101 in the Control group.

### Recruitment

Patients were enrolled in the study between May 2009 and April 2010. The last patient completed the trial in May 2010.

### Baseline data

The study population was found to be homogeneous with a mean age of 66 years old, BMI of 27.4 and a majority (68%) of female patients of ASA class I (27%) or II (58%). Patient characteristics seemed to be similar in the Sangvia and Control groups (Table 1). One clinic routinely used tranexamic acid, five patients received it, two in the Control group and three in the Sangvia group. All except four patients in the Sangvia group received autologous blood transfusion, collected either intra-operatively and/or postoperatively, and a mean of 522 mL was transfused (PP analysis).

**Table 1:** Patients characteristics.

Patients characteristics		Sangvia	Control	p-value^1^
	n	106	110	
Age (years)	Mean (SD)	67 (11)	65 (12)	0.1633
BMI	Mean (SD)	27.3 (4.6)	27.5 (4.6)	0.5097
Sex n (%)	Female	76 (72)	70 (64)	
	Male	30 (28)	40 (36)	0.2451
ASA n (%)	I	28 (26)	31 (28)	
	II	59 (56)	66 (60)	
	III	19 (18)	13 (12)	0.4017
Surgery n (%)	Primary	100 (94)	104 (95)	
	Revision	6 (6)	6 (6)	1.0000
Anesthesia n (%)	Spinal	63 (60)	63 (57)	
	General	40 (38)	42 (38)	
	Combined	2 (2)	5(5)	0.8255
Pre-op Hb (g/dL)	Mean (SD)	13.87 (1.16)	13.98 (1.16)	0.4736
Pre-op Hct (%)	Mean (SD)	41 (4)	42 (3)	0.3462

BMI = Body Mass Index.

1 Mann-Whitney U/Wilcoxon rank sum test: Exact Sig. (2-tailed) was used to give an indication of the size of chance imbalances between the treatment groups.

### Outcomes and estimation

**Primary outcome:** The ITT analysis showed a generally low allogeneic blood transfusion frequency of 14% in both groups (95% CI −0.09–0.10, p = 1.000) and efficacy was studied in the PP analysis where nine of 96 (9%) patients needed an allogeneic blood transfusion in the Sangvia group and 13 of 101 (13%) in the Control group. The 4% difference between the groups was not statistically significant (95% CI −0.05–0.12, p = 0.5016), the power for detecting it was 14%. A total of 15 transfusion decisions were taken for the nine patients transfused in the Sangvia group and 26 for the 13 patients transfused in the Control group (PP analysis). The transfusion volume among the patients receiving allogeneic blood was 756 mL (2.3 units), transfusion index = 2.33 in the Sangvia group and 856 mL (2.5 units), transfusion index = 2.54 in the Control group (PP analysis). Corresponding values from the ITT analysis were 24 transfusion decisions for 15 patients transfused, 735 mL (2.3 units) and transfusion index = 2.33 in the Sangvia group and 29 transfusion decisions for 15 patients transfused, 834 mL (2.6 units), transfusion index = 2.60 in the Control group. None of the differences measured were found to be statistically significant. Of the 30 patients that were transfused (53 transfusions), only 4 patients (5 transfusions) underwent revision surgery. Five transfusions were given during surgery without a known Hb value. For 28 transfusions the transfusion trigger of ≤8.5 g/dL was reached. The other 20 transfusions were given based on clinical symptoms. The transfusion percentage per center showed a large variance, ranging from 4% to 52% (4%, 9%, 11% twice, 20%, 52%).

**Secondary outcomes:** The results of the estimated and calculated blood loss are presented in Table 2. The PP analysis showed that the total estimated blood loss was 914 mL in the Sangvia group and 921 mL in the Control group (95% CI −131–117, p = 0.8798). Corresponding values from the ITT analysis were 931 mL in the Sangvia group and 927 mL in the Control group (95% CI −119–127, p = 0.9473). A smaller calculated blood loss was seen in the Sangvia group at days 2 and 4 compared to the Control group (PP analysis); 1145 mL vs. 1296 mL on day 2 (95% CI 17–285, p = 0.0633) and 1095 mL vs. 1285 mL on day 4 (95% CI 31–349, p = 0.0175). No early differences were seen at three hours (923 mL vs. 952 mL, 95% CI −147–89, p = 0.6671) or at day 1 (1104 mL vs. 1140 mL, 95% CI −159–87, p = 0.3845) after surgery. Corresponding values from the ITT analysis were 935 mL vs. 970 mL three hours after surgery (95% CI −155–85, p = 0.2512), 1081 mL vs. 1160 mL on day 1 (95% CI −199–41, p = 0.0665), 1148 mL vs. 1311 mL on day 2 (95% CI 36–290, p = 0.0129) and 1104 mL vs. 1284 mL on day 4 (95% CI 26–334, p = 0.0086).

**Table 2:** Estimated and calculated blood loss per treatment group.

		Sangvia	Control	95% CI, p-value^1^
**ITT analysis set**				
**Estimated blood loss (mL)**				
Intra-operative	Mean (SD)	479 (329)	517 (305)	−124–48, 0.2394
Postoperative 0–6 h	Mean (SD)	305 (188)	292 (182)	−40–66, 0.6960
Postoperative 6–24 h	Mean (SD)	220 (126)	212 (171)	−36–52, 0.1863
Total	Mean (SD)	931 (486)	927 (431)	−119–127, 0.9473
**PP analysis set**				
**Calculated blood loss (mL)**				
3 hours after surgery	Mean (SD)	923 (407)	952 (419)	−147–89, 0.6671
Postoperative day 1	Mean (SD)	1104 (418)	1140 (449)	−159–87, 0.3845
Postoperative day 2	Mean (SD)	1145 (436)	1296 (500)	17–285, 0.0633
Postoperative day 4	Mean (SD)	1095 (480)	1285 (562)	31–349, 0.0175

1 Mann-Whitney U/Wilcoxon rank sum test: Exact Sig. (2-tailed), 95% CI based on independent sample t-test, equal variances assumed.

The Hb and Hct values are presented in Table 3 as relative change from screening (PP analysis). For the Hb values, lower reduction was seen in the Sangvia group but no statistically significant differences were detected in a comparison of the two treatment groups. Corresponding values from the ITT analysis set were 2.53 g/dL vs. 2.58 g/dL at three hours (95% CI −0.35–0.25, p = 0.9629), 3.03 g/dL vs. 3.14 g/dL at day 1 (95% CI −0.40–0.18, p = 0.3222), 3.13 g/dL vs. 3.41 g/dL at day 2 (95% CI −0.57–0.01, p = 0.0741) and 3.05 g/dL vs. 3.30 g/dL at day 4 (95% CI −0.60–0.10, p = 0.1230).

**Table 3:** Hb and Hct change from screening per treatment group (PP analysis set).

		Sangvia	Control	95% CI, p-value^1^
**Hb (g/dL)**				
3 h after surgery	Mean (SD)	2.53 (0.98)	2.54 (1.12)	−0.31–029, 0.7345
1 day after surgery	Mean (SD)	3.11 (1.04)	3.10 (1.11)	−0.29–0.31, 0.8089
2 days after surgery	Mean (SD)	3.14 (1.06)	3.38 (1.06)	−0.54–0.06, 0.1302
4 days after surgery	Mean (SD)	3.06 (1.16)	3.31 (1.22)	−0.61–0.11, 0.1406
**Hct (%)**				
3 h after surgery	Mean (SD)	8 (3)	8 (3)	−1–1, 0.6873
1 day after surgery	Mean (SD)	9 (3)	9 (4)	−1–1, 0.8020
2 days after surgery	Mean (SD)	9 (3)	10 (4)	−2–0, 0.1404
4 days after surgery	Mean (SD)	9 (4)	10 (4)	−2–0, 0.0211

1 Mann-Whitney U/Wilcoxon rank sum test: Exact Sig. (2-tailed)), 95% CI based on independent sample t-test, equal variances assumed.

Regarding the change in Hct percentages, the reduction in the Sangvia group was significantly lower than in the Control group on day 4 (p = 0.02; 95% CI −2–0). Corresponding values from the ITT analysis set were 8% vs. 8% at three hours (95% CI −1–1, p = 0.9958), 9% vs. 9% at day 1 (95% CI −1–1, p = 0.3370), 9% vs. 10% at day 2 (95% CI −2–0, p = 0.0676) and 9% vs. 10% at day 4 (95% CI −2–0, p = 0.0169).

Laboratory parameters and vital signs showed no differences in overall heart rate and temperature between the two treatment groups at any time point (ITT analysis). The blood pressure measurements however indicated that there was a smaller reduction in blood pressure during surgery in patients in the Sangvia group, i.e. difference of 8 mmHg in systolic blood pressure (95% CI 4–12, p = 0.0046*)* and 6 mmHg in diastolic blood pressure (95% CI 3–9, p = 0.0006*)*, as compared to the Control group (ITT analysis). The difference in diastolic blood pressure also seemed to persist on day 1 (difference 3 mmHg: 95% CI 0–6, p = 0.0273), day 2 (difference 5 mmHg: 95% CI 1–9, p = 0.0098), day 3 (difference 6 mmHg: 95% CI 2–10, p = 0.0053) and day 4 (difference 8 mmHg, 95% CI 3–13, p = 0.0012) after surgery (ITT analysis). All mean/median values for sodium, potassium, creatinine and Glomerular Filtration Rate were within normal reference intervals of 135–145 mmol/L, 3.5–5.0 mmol/L, 62–95 µmol/L and 55–134 mL/min/1.73 m^2^, respectively, and no differences were detected between the groups at any time point (ITT/PP analysis).

The assessment with the EQ-5D questionnaire showed an expected general improvement in mobility, self-care, usual activity, pain and anxiety in both groups (ITT analysis). After two months, problems in mobility, self-care, usual activity, pain and anxiety were reported for 48%, 28%, 38% 46% and 10%, respectively, in the Sangvia group and for 52%, 27%, 54%, 47% and 10%, respectively, in the Control group. The median general improvement in health status was from 70 to 80 on the Visual Analogue Scale (VAS) in both groups.

### Adverse events

Forty three of 106 (41%) patients in the Sangvia group and 46 of 110 (42%) patients in the Control group had one or more adverse events (difference 1%: 95% CI −0.14–0.12, p = 0.8905), leading to a total of 141 adverse events. The numbers of patients with one, two or three reported adverse events were coded and are compared in Table 4. Twelve patients (11%) in the Sangvia group reported 14 adverse events that were classified as either possibly or probably/definitely device related. The following adverse events were classified as possibly related: 1× anaemia, 2× headache/vertigo/nausea, 2× pain during transfusion, 2× seroma, 2× wound leakage, 1× wound swelling, 1× hematuria, 1× saturation depression and 1× high heart beat. In addition, one reported wound leakage was classified as probably/definitely device related.

**Table 4:** All (serious) adverse events coded according to WHO-ART.

		Sangvia	Control
N		106	110
Adverse events per system-organ class^1^	N events		
Skin and appendages disorders	1	1	1
Musculo-skeletal system disorders	1	3	1
Central & peripheral nervous system disorders^2^	1	7	1
	2	1	0
Psychiatric disorders	1	1	3
Gastro-intestinal system disorders	1	8	7
	2	4	3
Metabolic and nutritional disorders	1	3	0
Cardiovascular disorders, general	1	4	9
Heart rate and rhythm disorders	1	3	2
Respiratory system disorders	1	3	2
Red blood cell disorders	1	2	3
Platelet, bleeding and clotting disorders	1	0	1
Urinary system disorders	1	3	3
Body as a whole - general disorders^3^^,^ 4	1	13	18
	2	4	1
	3	1	1
Resistance mechanism disorders	1	3	4

Number of patients with 1, 2 or 3 reported adverse events per system organ class.

1 System organ class according to WHO Adverse Reaction Terminology (WHO-ART) was used for coding by means of Primary System according to the Adverse Event Dictionary Version 029 (equivalent to MedDRA).

2 Reported AEs were dizziness, headache, nausea, myoclonus, vertigo, restless legs, and needling sensation during transfusion.

3 Body as a whole – general disorders include for example postoperative complications (e.g. wound seroma and/or redness and hip joint dislocation), peripheral edema, pain and death.

4 There was one reported death in the Control group, which occurred 13 days after discharge.

Seven serious adverse events occurred in both groups (difference 1%, 95% CI −0.06–0.07, p = 1.000). One patient in the Control group died 13 days after surgery. All other events were serious due to prolongation of hospitalization. Table 5 list all serious adverse events per treatment group collected in the study. To further explore the correlation between adverse events and autologous blood transfusion special attention was paid to reported adverse events among patients with the highest transfusion volumes in the Sangvia group (i.e. 75% percentile, representing transfusion volume>669 mL). These are listed in Table 6. One reported serious adverse event (i.e. vasovagal episode) was found in one patient with a total transfusion volume of 700 mL. No indications were otherwise seen of more severe adverse events with increasing transfusion volume.

**Table 5:** Reported serious^1^ adverse events.

Adverse event	Sangvia	Control
Cardiac insufficiency	1	0
Dehydration	1	0
Hip dislocation/luxation	1	1
Lung embolism	0	1
Paralytic ileus	1	0
Periprosthetic fissure (intra-op)	0	1
Saturation depression	1	0
Death	0	1
Infection hip	0	1
Suspected infection (positive bacterial culture)	1	0
Wound infection	0	1
Wound leakage	1	1
Total	7	7

1 Serious definition according to ICH/Good Clinical Practice as any untoward and unintended response that results in death, is immediately life-threatening, requires in-subject hospitalization or prolongation of existing hospitalization, results in persistent or significant disability or incapacity, is a congenital abnormality or birth defect or is an important medical event that may jeopardize the patient or may require medical intervention to prevent one of the outcomes listed above.

**Table 6:** Reported adverse events for Sangvia group with autologous transfusion volume>669 mL^1^.

Transfusion volume (mL) per patient	Number of patients	Number of AEs per patient	AE specification
675	1	0	
700	4	0	
700	2	1	Needling stings in skin during blood transfusion
			Delirium
700	1	2	Nausea
			Vomiting
700	1	4	Anterior cortical femur fracture
			Dizziness and light-headedness
			Hip dislocation
			Vasovagal episode
725	1	0	
750	1	0	
750	1	1	Leg pain 3 weeks after surgery
800	1	0	
825	1	0	
850	1	3	Oedema of scrotum and both legs
			Seroma
			Three small wounds (1×1 cm), circulatory disorder
900	2	0	
950	2	0	
1000	1	0	
1050	2	1	Pain in the leg
			Anaemia bleeding
1050	1	3	Headache and nausea
			Vertigo and nausea
			Vomiting
1300	1	0	
1400	1	2	Abscess perianal with purulent secretion
			Seroma
2720	1	3	Hematuria
			Muscle cramp in upper thigh
			Wound leakage

1 The Sangvia transfusion volume was divided by percentile, i.e. 25% = 306 mL, 50% = 475 mL and 75% = 669 mL.

## Discussion

### Interpretation

In our assessor–blinded, randomized, controlled, parallel-group trial that included 216 patients, a low use of allogeneic blood was seen when the Sangvia™ Blood Management System was used together with a transfusion protocol. The trial setting was not able to verify efficacy with regards to allogeneic transfusion frequency for the system itself, but a lower calculated blood loss and lower hematocrit reduction was seen four days after surgery among patients receiving autologous blood. The study contributes descriptive data on safety, and no safety issues were discovered with the use of the new device. Further large-scale, randomized, controlled trials are warranted to continue to investigate the safety and efficacy of the device.

Carless et al. [17] conducted a systematic review to investigate the effectiveness of cell salvage in orthopedic, cardiac and vascular surgery. Overall, the findings showed that cell salvage reduces the need for transfusions of donated blood in cardiac and orthopedic surgery. These conclusions were drawn with the remark that the methodological quality of the trials was poor and that the findings may be biased in favor of cell salvage [17]. This is why large trials of high methodological quality that assess the relative effectiveness, safety and cost-effectiveness of cell salvage are necessary. Although the quality of the current study design was strengthened by using an independent and blinded transfusion trigger assessor, we cannot rule out potential bias as allogeneic transfusions were also allowed for clinical symptoms and transfusion decisions were taken by clinicians aware of treatment allocation in acute situations during surgery. Blinding is a cornerstone of therapeutic assessment to mitigate the risk of bias and previous studies have shown larger treatment effects in cases of un-blinded endpoint assessment [30]. However, blinding patients, care providers and outcome assessors is difficult to achieve when surgery techniques are being studied, and unblinding may thus occur more often in such studies [31]. The results of the current study, based on blinded laboratory analyses, show some efficacy benefits with lower calculated blood loss and hemaocrit reduction, supporting the use of cell salvage. However, efficacy with regard to allogeneic blood transfusion needs to be further verified in a larger trial setting.

Our study seems to confirm previously reported safety results with the Sangvia™ Blood Management System [20], [21] and proposes that it might be safe to use in orthopedic surgery even though it is recognized that the study was not primarily powered for safety conclusions. This speculation is based on the fact that laboratory variables collected for safety analysis did not show any differences between the two groups and all stayed within the reference ranges, and that the majority of reported adverse events were non-severe. In addition, no general safety issues were raised when investigating reported adverse events and both treatment groups had very similar adverse event profiles, as shown in Table 4. Furthermore, no indications were seen of more severe adverse events with increasing transfusion volume when examining the adverse events reported from patients that received the highest transfusion volumes with Sangvia, as shown in Table 6.

### Limitations and Generalizability

Our study is strengthened by using an independent and blinded transfusion trigger assessor. Blinding is a cornerstone of therapeutic assessment to mitigate the risk of bias. For instance, previous studies have shown larger treatment effects in cases of un-blinded endpoint assessment [30]. Blinding patients, care providers and outcome assessors when assessing a non-pharmacological trial is more difficult than in pharmacological trials, which is why blinding is not always appropriate and unblinding may occur more often [31].

The primary limitation of the study relates to low power since an adaptive interim analysis concluded that the allogeneic transfusion rate in the Control group was much lower (12%) than the expected 21% and the study was prematurely discontinued. The results of the PP analysis showed a transfusion rate of 9% in the Sangvia group and 13% in the Control group, indicating a 4% difference between the groups (95% CI −0.05–0.12, p = 0.5016). This difference was not statistically verified, however, and the power for detecting it, if true, was only 14%. A new study, performed in the same trial setting and having the aim to detect a potential difference at the 4% level, with a power of 90% and a two-sided level of significance of 5%, would require at least 2572 patients. The ITT analysis did not indicate any differences in transfusion rate between the treatment groups (14% in both groups, 95% confidence interval −9–10, p = 1.000). However, this analysis set should be used with care when drawing efficacy conclusions because it included patients with major protocol deviations (e.g. Sangvia was used in the Control group and epoetin alfa and other autologous blood transfusion were used with a potential impact on the need for allogeneic blood transfusion).

As described in the participant flow in the results section it is worth to mention once again that the data presented in the ITT analysis was limited to all treated patients due to missing follow-up data for patients excluded before treatment allocation. It is recognized that this is not per definition a formal ITT analysis since it should include all patients intended for treatment, for our study that means also the ones that did not make it into the operating room. Thus, our study may have skewed results due to post-randomisation bias. This illustrates the more complicated nature of surgical randomized trials and stresses the need for randomization as close as possible to the intervention or control treatment preventing this limitation in our study and subsequent difficulties in analysing the results using formal ITT analysis.

Accordingly, any potential differences in the efficacy of the intervention would be weakened and unlikely to be discovered in the ITT analysis set owing to the low power of the study. However, the ITT is interesting in exploring the effectiveness of the treatment, although it is difficult to relate to the reason why there are differences in the results of the ITT and PP analyses, i.e. they may either relate to poor treatment efficacy or poor treatment implementation.

The low allogeneic transfusion frequency found in the study affects the generalizability of the results. First, the literature refers to the use of transfusion trigger protocols in transfusion medicine but it is likely that this was used more strictly in the current clinical trial setting than in normal practice based on the facts that an assessor-blinded study design was used and allogeneic transfusion frequencies found in the literature [12], [26] were much higher than those observed in the study. Second, the study population was homogeneous with regard to demographic and baseline variables but generally healthier than the expected target population of the study. For example, the patients were young (mean age of 66 years) and healthy (ASA class I in 27% of the cases) and very few revision hip arthroplasties were included (only 6%). The latter was prominent in one clinic that primarily included ASA class I patients with high pre-operative Hb levels since the epoetin alfa guideline at the hospital restricted the inclusion of patients with lower Hb levels. Third, there were some indications of selection bias toward uncomplicated surgery in the study. For example, no revision hip arthroplasties were included at one clinic. In summary, the present results were based on a population that was challenging in terms of studying efficacy improvements.

Further limitations of the study relate to the generalizability of the results. For instance, the inclusion and exclusion criteria regarding hemoglobin values were very strict, i.e. a Hb level above 11 g/dL and no use of epoetin alfa. The latter excluded patients with a pre-operative hemoglobin value between 11 g/dL and 13 g/dL in centers using a routine regimen of epoetin alfa. Salido et al. [32] showed that pre-operative hemoglobin values have a predictive value for the need for allogeneic blood transfusions why it could be expected to be easier to detect efficacy differences in centers without implemented blood management programs. Furthermore, some patients were excluded from the PP analysis as a result of poor protocol implementation, e.g. other autologous blood devices and erythropoietin were used by mistake. Although study personnel were trained before the study was started, it can be concluded that this was not sufficient to avoid major protocol deviations in the study. This was especially true for clinics that had to change their normal practice to adapt to the standardized clinical study protocol. More emphasis should thus be placed on training before initiating future clinical studies in this area.

### Overall evidence

Our study was not able to draw general conclusions on efficacy, but the safety data propose that the Sangvia™ Blood Management System may be safe to use in orthopedic surgery. Both these aspects need to be further investigated in large-scale clinical research. It could also be interesting to compare the cost-effectiveness with other therapy options. For instance, a comparison could be made with a preoperative alternative such as epoetin alfa in a non-inferiority design. Epoetin alfa is paid in most countries by other health care resources than the hospital budget, and thus cost effectiveness should focus on the whole health care community. It would also be interesting to conduct a clinical efficacy trial for the Sangvia™ Blood Management System in patients with a higher allogeneic transfusion rate risk, e.g. revision surgery, and/or in patients with low pre-operative Hb values, with a special focus on the methodological quality [17], [33], and as utilized in the presented study.

## Supporting Information

Checklist S1 **CONSORT checklist.**
(DOC)

Protocol S1 **Trial protocol.**
(PDF)

## References

[1] Grzelak I, Zaleska M, Olszewski WL (1998) Blood transfusions downregulate hematopoiesis and subsequently downregulate the immune response. Transfusion 38: 1104–1114. 9838944 10.1046/j.1537-2995.1998.38111299056323.x

[2] Tartter PI (1995) Immunologic effects of blood transfusion. Immunol Invest 24: 277–288. 7713588 10.3109/08820139509062778

[3] Innerhofer P, Klingler A, Klimmer C, Fries D, Nussbaumer W (2005) Risk for postoperative infection after transfusion of white blood cell-filtered allogeneic or autologous blood components in orthopedic patients undergoing primary arthroplasty. Transfusion 45: 103–110. 15647025 10.1111/j.1537-2995.2005.04149.x

[4] Duffy G, Neal KR (1996) Differences in post-operative infection rates between patients receiving autologous and allogeneic blood transfusion: a meta-analysis of published randomized and nonrandomized studies. Transfus Med 6: 325–328. 8981727 10.1111/j.1365-3148.1996.tb00091.x

[5] Dodd RY (2005) Current estimates of transfusion safety worldwide. Dev Biol (Basel) 120: 3–10. 16050149

[6] Wylie BR (1993) Transfusion transmitted infection: viral and exotic diseases. Anaesth Intensive Care 21: 24–30. 8447602 10.1177/0310057X9302100109

[7] Glance LG, Dick AW, Mukamel DB, Fleming FJ, Zollo RA, et al (2011) Association between intraoperative blood transfusion and mortality and morbidity in patients undergoing noncardiac surgery. Anesthesiology 114: 283–292. 21239971 10.1097/ALN.0b013e3182054d06

[8] Spahn DR, Moch H, Hofmann A, Isbister JP (2008) Patient blood management: the pragmatic solution for the problems with blood transfusions. Anesthesiology 109: 951–953. 19034088 10.1097/ALN.0b013e31818e3d75

[9] Shander A, Hofmann A, Ozawa S, Theusinger OM, Gombotz H, et al (2010) Activity-based costs of blood transfusions in surgical patients at four hospitals. Transfusion 50: 753–765. 20003061 10.1111/j.1537-2995.2009.02518.x

[10] Thoms RJ, Marwin SE (2009) The role of fibrin sealants in orthopaedic surgery. J Am Acad Orthop Surg 17 10.5435/00124635-200912000-00001 19948697

[11] Sukeik M, Alshryda S, Haddad FS, Mason JM (2011) Systematic review and meta-analysis of the use of tranexamic acid in total hip replacement. J Bone Joint Surg Br 93: 39–46. 21196541 10.1302/0301-620X.93B1.24984

[12] Weber EW, Slappendel R, Hemon Y, Mahler S, Dalen T, et al (2005) Effects of epoetin alfa on blood transfusions and postoperative recovery in orthopaedic surgery: the European Epoetin Alfa Surgery Trial (EEST). Eur J Anaesthesiol 22: 249–257. 15892401 10.1017/s0265021505000426

[13] Moonen AF, Knoors NT, van Os JJ, Verburg AD, Pilot P (2007) Retransfusion of filtered shed blood in primary total hip and knee arthroplasty: a prospective randomized clinical trial. Transfusion 47: 379–384. 17319816 10.1111/j.1537-2995.2007.01127.x

[14] Munoz M, Ariza D, Garceran MJ, Gomez A, Campos A (2005) Benefits of postoperative shed blood reinfusion in patients undergoing unilateral total knee replacement. Arch Orthop Trauma Surg 125: 385–389. 15821894 10.1007/s00402-005-0817-3

[15] Munoz M, Garcia-Erce JA, Villar I, Thomas D (2009) Blood conservation strategies in major orthopaedic surgery: efficacy, safety and European regulations. Vox Sang 96: 1–13. 10.1111/j.1423-0410.2008.01108.x 19121192

[16] Garvin KL, Feschuk CA, Sekundiak TD, Lyden ER (2005) Blood salvage and allogenic transfusion needs in revision hip arthroplasty. Clin Orthop Relat Res 441: 205–209. 16331004 10.1097/01.blo.0000192033.50316.bf

[17] Carless PA, Henry DA, Moxey AJ, O'Connell D, Brown T, et al (2010) Cell salvage for minimising perioperative allogeneic blood transfusion. Cochrane Database Syst Rev CD001888 10.1002/14651858.CD001888.pub2 17054147

[18] Munoz M, Cobos A, Campos A, Ariza D, Munoz E, et al (2006) Post-operative unwashed shed blood transfusion does not modify the cellular immune response to surgery for total knee replacement. Acta Anaesthesiol Scand 50: 443–450. 16548856 10.1111/j.1399-6576.2006.00977.x

[19] Andersson I, Tylman M, Bengtson JP, Bengtsson A (2001) Complement split products and pro-inflammatory cytokines in salvaged blood after hip and knee arthroplasty. Can J Anaesth 48: 251–255. 11305825 10.1007/BF03019754

[20] Kvarnstrom A, Schmidt A, Tylman M, Jacobsson M, Bengtsson A (2008) Complement split products and proinflammatory cytokines in intraoperatively salvaged unwashed blood during hip replacement: comparison between heparin-coated and non-heparin-coated autotransfusion systems. Vox Sang 95: 33–38. 18444947 10.1111/j.1423-0410.2008.01059.x

[21] Stachura A, Krol R, Poplawski T, Michalik D, Pomianowski S, et al (2011) Transfusion of intra-operative autologous whole blood: influence on complement activation and interleukin formation. Vox Sang 100: 239–246. 21118266 10.1111/j.1423-0410.2010.01377.x

[22] Hurle R, Poma R, Maffezzini M, Manzetti A, Piccinelli A, et al (2004) A simple mathematical approach to calculate blood loss in radical prostatectomy. Urol Int 72: 135–139. 14963354 10.1159/000075967

[23] del Trujillo MM, Carrero A, Munoz M (2008) The utility of the perioperative autologous transfusion system OrthoPAT in total hip replacement surgery: a prospective study. Arch Orthop Trauma Surg 128: 1031–1038. 17828546 10.1007/s00402-007-0440-6

[24] EuroQol–a new facility for the measurement of health-related quality of life. The EuroQol Group. Health Policy 16: 199–208. 10109801 10.1016/0168-8510(90)90421-9

[25] Brooks R (1996) EuroQol: the current state of play. Health Policy 37: 53–72. 10158943 10.1016/0168-8510(96)00822-6

[26] Grosvenor D, Goyal V, Goodman S (2000) Efficacy of postoperative blood salvage following total hip arthroplasty in patients with and without deposited autologous units. J Bone Joint Surg Am 82-A: 951–954. 10901309 10.2106/00004623-200007000-00006

[27] Strumper D, Weber EW, Gielen-Wijffels S, Van DR, Bulstra S, et al (2004) Clinical efficacy of postoperative autologous transfusion of filtered shed blood in hip and knee arthroplasty. Transfusion 44: 1567–1571. 15504161 10.1111/j.1537-2995.2004.03233.x

[28] Weber EW, Slappendel R, Prins MH, van der Schaaf DB, Durieux ME, et al (2005) Perioperative blood transfusions and delayed wound healing after hip replacement surgery: effects on duration of hospitalization. Anesth Analg 100: 1416–21. 15845698 10.1213/01.ANE.0000150610.44631.9D

[29] Lachin JM (1981) Introduction to sample size determination and power analysis for clinical trials. Control Clin Trials 2: 93–113. 7273794 10.1016/0197-2456(81)90001-5

[30] Poolman RW, Struijs PA, Krips R, Sierevelt IN, Marti RK, et al (2007) Reporting of outcomes in orthopaedic randomized trials: does blinding of outcome assessors matter? J Bone Joint Surg Am 89: 550–558. 17332104 10.2106/JBJS.F.00683

[31] Boutron I, Tubach F, Giraudeau B, Ravaud P (2004) Blinding was judged more difficult to achieve and maintain in nonpharmacologic than pharmacologic trials. J Clin Epidemiol 57: 543–550. 15246122 10.1016/j.jclinepi.2003.12.010

[32] Salido JA, Marin LA, Gomez LA, Zorrilla P, Martinez C (2002) Preoperative hemoglobin levels and the need for transfusion after prosthetic hip and knee surgery: analysis of predictive factors. J Bone Joint Surg Am 84-A: 216–220. 11861727 10.2106/00004623-200202000-00008

[33] Sharma R, Farrokhyar F, McKnight LL, Bhandari M, Poolman RW, et al (2011) Quality of assessment of randomized controlled trials in blood conservation after joint arthroplasty. J Arthroplasty 26: 909–913. 21074359 10.1016/j.arth.2010.08.014

