# Supplementary material for: Limit Allogeneic Blood Use with Routine Re-use of Patient's Own Blood: A Prospective, Randomized, Controlled Trial in Total Hip Surgery
Source: PLoS One. 2012 Sep 13;7(9):e44503. doi: 10.1371/journal.pone.0044503 (PMC3441549; doi:10.1371/journal.pone.0044503)
Supplement: Protocol S1 — Trial protocol. (PDF) [file pone.0044503.s002.pdf]

A company in the AstraZeneca Group

ASTRA
ASTRA TECH

CLINICAL STUDY PROTOCOL

Study Product:	Sangvia®
Study Code:	YA-DRA-0001
Edition No.:	Final 2.0
Date:	12th January 2009

A prospective, randomized, controlled trial of retransfusion of intra-operatively collected filtered whole blood in total hip surgery

Sponsor:
Astra Tech AB
Aminogatan 1
SE-432 21 Mölndal, Sweden.

The following Amendment(s) and Administrative Changes have been made to this protocol since the date of preparation:

Amendment No.	Date of Amendment	Local Amendment No.	Date of Local Amendment

Administrative Change No.	Date of Administrative Change	Local Administrative Change No.	Date of Local Administrative Change

This submission /document contains trade secrets and confidential commercial information, disclosure of which is prohibited without providing advance notice to ASTRA TECH AB and opportunity to object.

ASTRA
ASTRA TECH

PROTOCOL SYNOPSIS

International Coordinating Investigator:
Dr. Rudolf W. Poolman, Onze Lieve Vrouwe Gasthuis (OLVG), Amsterdam

Study centres and investigator

Centre No.	Centre	Principal Investigator
1	Onze Lieve Vrouwe Gasthuis (OLVG)<br>1e Oosterparkstraat 279/<br>1091 HA Amsterdam<br>Postbus 95500/1090 HM Amsterdam<br>Netherlands	Dr. Rudolf W. Poolman<br>Tel: +31 205999111<br>Mail: r.w.poolman@olvg.nl
2	MC Haaglanden<br>Orthopedic Dept.<br>P.O. Box 432<br>2501 CK The Hague<br>The Netherlands	Drs. Bregje J.W. Thomassen<br>Tel: +31 703303109<br>Mail: b.thomassen@mchaaglanden.nl
3	Reinier de Graaf Gasthuis (RdGG)<br>afd. Orthopedie<br>Pb 5501<br>2600 GA Delft<br>Netherlands	Dr. Peter Pilot<br>Tel: +31 152603257<br>Mail: Pilot@rdgg.nl
4	Hospital Universitario Mar- Esperança (IMAS)<br>Servicio de Anestesiología<br>Passeig marítim, 25<br>08003 Barcelona<br>Spain	Dra. Elvira Bisbe<br>Tel: +34 616 664224<br>Mail: Ebisbe@imas.imim.es
5	St. Olavs Hospital<br>Ortopedisk avdelning<br>Olav Kyrres gate 17<br>7006 Trondheim<br>Norway	Dr. Ketil Holen<br>Tel: +4772574394<br>Mail: ketil.holen2@stolav.no
6	Medical University Vienna<br>Department of Orthopaedic Surgery<br>Währinger Gürtel 18-20<br>1090 Wien<br>Austria	Dr. Josef Grohs<br>Tel: +431404004082<br>Mail:josef.grohs@meduniwien.ac.at

Competitive recruitment of a total of 300 patients and a minimum of 20 patients per clinic will be applied.

**Study timetable**		**Phase of development**
Estimated date of first subject enrolled	28th February 2009	Post-marketing
Estimated date of last subject completed	30th November 2009	

Objectives

The primary objective of this study is to compare clinical efficacy in total hip replacement patients receiving either blood transfusion with the Sangvia® Blood Management System or no autologous transfusion by assessment of need for allogeneic blood transfusion.

The secondary objectives of the study are….

….to observe and compare clinical efficacy by means of postoperative infection rates, use of postoperative antibiotics, length of hospital stay and health status for patients transfused with Sangvia® blood, no blood and/or allogeneic blood.

….to observe and compare clinical efficacy by means of systemic postoperative haemoglobin levels for patients transfused with Sangvia® blood, no blood or allogeneic blood.

…. to collect details in health care utilisation (medications, in-patient service use, blood management) that would allow a calculation and comparison of cost-consequences applying either alternative in the blood management and from the perspective of the payer

…..to observe and compare safety by means of adverse events, highlighting renal failures and events potentially related to transfusion, body temperature and other clinical complications for patients transfused with Sangvia® blood, no blood or allogeneic blood.

Study design

The study is an assessor blind, prospective, randomized, controlled, multi-centre investigation of 300 patients.

Target subject population

Patients scheduled for either primary or secondary total hip arthroplasty

Investigational product

Intra-operative, and if bleeding continues after surgery, possibly also post-operative autologous blood transfusion with the Sangvia® system (test group).

Comparator

No autologous blood, i.e. allogeneic blood transfusion when needed (control group).

Duration of treatment

Patients will be followed during their hospital stay and at 2 months after discharge.

Outcome variables

Primary outcome variable:

- Frequency and amount of allogeneic blood transfusion

Secondary outcome variable(s):

- Post-operative infection rate (SIRS reaction, clinical symptoms, wound infection, CRP and LPC)
- Post-operative antibiotic use
- Length of hospital stay
- Post-operative haemoglobin concentration
- Demographical data (e.g. vital signs, age, gender etc)
- Health status questionnaire (i.e. EQ-5D)

Safety variable(s):

- Adverse events (AE, SAE, ADE) rated for causality
- Laboratory measurements (Hb, Hct/EVF, TPC, LPC, Na^+, K^+, CRP, Creatinine and Glomerular Filtration Rate (GFR)/Creatinine Clearance (C_{Cr}))

Health economics variable(s):

- Units and ml of allogeneic blood transfusions
- Number of post-operative bed days by general and intensive care unit
- Post-operative medications

Statistical methods

Descriptive statistics, i.e. mean, median, standard deviation, minimum and maximum values for continuous data and frequencies and percentages for categorical data, will be used together with different statistical tests. Fischer Exact test will be used to test frequencies of dichotomous response variables. The non- parametric Wilcoxon Rank Sum test will be used to test differences in continuous response variables between the parallel treatment groups. Wilcoxon Signed Rank test will be used to test differences over time within each treatment group. P-values equal to or below 0.05 are considered as statistically significant. Subgroup analyses of type (primary and secondary) and complexity (easy < 1 hour, medium 1-2 hours and complex > 2 hours) of surgery are planned.

TABLE OF CONTENTS PAGE

1 INTRODUCTION 9
1.1 Background 9
1.2 Rationale 10
2 STUDY OBJECTIVES 10
2.1 Primary objective 10
2.2 Secondary objectives 10
3 STUDY PLAN AND PROCEDURES 11
3.1 Overall study design and flow chart 11
3.2 Rationale and risk/benefit assessment 17
3.2.1 Risk/benefit assessment 18
3.2.2 Ethical considerations 18
3.3 Selection of study population 19
3.3.1 Study selection record 19
3.3.2 Inclusion criteria 19
3.3.3 Exclusion criteria 19
3.3.4 Restrictions 20
3.3.5 Discontinuation of subjects from treatment or assessment 20
3.3.5.1 Criteria for discontinuation 20
3.3.5.2 Procedures for discontinuation 20
3.3.5.3 Procedures for handling incorrect enrolled subjects 20
3.4 Treatments 21
3.4.1 Identity of investigational product and comparators 21
3.4.2 Treatment regimens 22
3.4.3 Labeling 22
3.4.4 Storage 22
3.4.5 Accountability 22
3.5 Method of assigning subjects to treatment groups 23
3.6 Blinding and procedures for unblinding the study 23
3.6.1 Methods for ensuring blinding 23
3.6.2 Methods for unblinding the study 23
3.7 Pre-study, concomitant and post-study treatment(s) 24
3.8 Treatment compliance 24
4 MEASUREMENTS OF STUDY VARIABLES AND DEFINITIONS OF OUTCOME VARIABLES 24
4.1 Screening and demographic measurements 24
4.2 Primary outcome variable 25
4.2.1 Allogeneic blood transfusion 25
4.2.1.1 Methods of assessment 25
4.2.1.2 Derivation or calculation of variable 25
4.3 Secondary outcome variable 25
4.3.1 Post-operative infection rate 25
4.3.1.1 Methods of assessment 25
4.3.1.2 Derivation or calculation of variable 26
4.3.2 Post-operative antibiotics use 26
4.3.2.1 Methods of assessment 26
4.3.2.2 Derivation or calculation of variable 26

4.3.3 Length of hospital stay 26
4.3.3.1 Methods of assessment 26
4.3.3.2 Derivation or calculation of variable 26
4.3.4 Post-operative Hb 26
4.3.4.1 Methods of assessment 26
4.3.4.2 Derivation or calculation of variable 27
4.4 Safety measurements and variables 27
4.4.1 Adverse Events 27
4.4.1.1 Definitions 27
4.4.1.2 Recording of Adverse Events and Adverse Device Effects. 28
4.4.1.3 Reporting of Serious Adverse Events 28
4.5 Patient-Reported Outcomes (PROs) 29
4.5.1 Health status questionnaire 29
4.5.1.1 Methods of assessment 29
4.5.1.2 Derivation or calculation of variable 29
4.6 Health Economic measurements and variables 29
4.6.1 Health care utilisation 29
4.6.1.1 Methods of assessment 29
4.6.1.2 Derivation or calculation of variable 29
4.7 Volume of blood sampling and handling of biological samples 29
4.7.1 Analysis of biological samples 30
4.7.1.1 Clinical chemistry samples 30
5 DATA MANAGEMENT 30
6 STATISTICAL METHODS AND DETERMINATION OF SAMPLE SIZE 31
6.1 Statistical evaluation – general aspects 31
6.1.1 Demographics and other baseline characteristics 31
6.1.2 Covariates and prognostic variables 31
6.1.3 Handling of dropouts and missing data 31
6.1.4 Multi-centre 31
6.2 Description of outcome variables in relation to objectives and hypotheses. 32
6.3 Description of analysis sets 32
6.4 Method of statistical analysis 32
6.5 Determination of sample size 33
6.6 Statistical analyses during the course of the study 33
7 STUDY MANAGEMENT 34
7.1 Monitoring 34
7.2 Audits and inspections 34
7.3 Training of staff 35
7.4 Changes to the protocol 35
7.5 Study agreements 35
7.6 Study timetable 35
8 ETHICS 36
8.1 Ethics review 36
8.2 Ethical conduct of the study 36
8.3 Informed consent 36
8.4 Subject data protection 37
9 PROCEDURES IN CASE OF EMERGENCY 37
9.1 Medical emergency 37
9.2 Pregnancy 37
10 REFERENCES 39

LIST OF TABLES PAGE

Table 1 Study plan 15
Table 2 Volume of blood to be drawn from each subject 29

LIST OF FIGURES PAGE

Figure 1 Study flow chart 14

LIST OF APPENDICES

Appendix A Signatures
Appendix B Investigators and Study Administrative Structure

LIST OF ABBREVIATIONS AND DEFINITION OF TERMS

The following abbreviations and special terms are used in this study protocol.

Abbreviation or special term	Explanation
ADE	Adverse Device Effect (see definition in Section 4.4.1.1)
AE	Adverse Event (see definition in Section 4.4.1.1)
C_{Cr}	Creatinine Clearance
CRF	Case Report Form
CRP	C-reactive protein
CSA	Clinical Study Agreement
Ethics Committee	Synonymous to Institutional Review Board and Independent Ethics Committee
EVF	Erythrocyte volume fraction, haematocrit
GCP	Good Clinical Practice
GFR	Glomerular Filtration Rate
Hb	Haemoglobin
Hct	Haematocrit, erythrocyte volume fraction,
International Co-ordinating investigator	If a study is conducted in several countries the International Co-ordinating Investigator is the Investigator co-ordinating the investigators and/or activities internationally.
ISF	Investigator's Study File
K^+	Potassium
LPC	Leukocyte Particle Count
Na^+	Sodium
OC	Oracle Clinical, the clinical data management system used at Astra Tech for capture and validation of study data
PRO	Patient Reported Outcome
Principal Investigator	A person responsible for the conduct of a clinical study at an investigational study site. Every investigational study site has a principal investigator
SAE	Serious adverse event (see definition in Section 4.4.1.1)
SIRS	Systemic Inflammatory Response Syndrome
TPC	Thrombocyte Particle Count/Platelets

1 INTRODUCTION

There are risks related to blood incompatibility and blood-borne diseases when using allogeneic transfusion during and after surgery. Several autologous transfusion alternatives exists on the market today and one of them, used for intra- and postoperative blood salvage, is the Sangvia® Blood Salvage System which has been proven safe to use in man. This study is designed to prove clinical benefits/efficacy of the Sangvia® system and to confirm previous reported safety results.

1.1 Background

Allogeneic blood transfusions subject patients to risk of infection, allergic reactions, immunosuppression and are also associated with high costs.[1-4] A variety of alternatives to allogeneic blood transfusions have been proposed, such as the use of solutions to support oxygen transport, human recombinant erythropoietin, intra-operative autologous blood collection, normovolemic hemodilution, preoperative blood salvage and postoperative infusion of shed wound blood.[5-11] At present autotransfusion is the most commonly used alternative to allogeneic blood transfusions and the application of the autotransfusion techniques has increased[12-14] along with a reduced need of allogeneic transfusion, reduced costs and minimized exposure to infectious agents. Although autologous blood is proven to be a safe transfusion product it represents only a small percentage of the total amount of blood transfused.
A number of autotransfusion devices are commercially available.[15,16] The techniques of peri-operative autotransfusion can be subdivided depending on the extent to which the scavenged blood is processed prior to transfusion. In “filtered autotransfusion systems”, the blood is often anticoagulated and filtered, whereas “washed autotransfusion systems” use anticoagulation, cell separation, washing and filtration. Filtered autotransfusion systems collect and transfuse whole blood. Blood is collected from the surgical wound through a suction device or drain and actively aspirated to a reservoir. This reservoir has a macro filter (e.g. 20-150 um) to remove large particles and cellular debris from the blood. A regulated negative pressure of no more than 150 mmHg is recommended to provide the suction necessary during blood collection. Larger negative pressures are believed to increase haemolysis. If applicable, the aspirated blood is anticoagulated either at the point of collection or in the reservoir. Even without anticoagulation, the blood collected for postoperative autotransfusion does not usually clot as it is defibrinogenated. The blood should be transfused within 6 hours of collection to preserve cellular viability and reduce the risk of infection.
There are several indications for autotransfusion, i.e.orthopaedic surgery,[15-17] vascular surgery,[18,19] liver transplantation,[20] trauma[21,22] and cardiac surgery.[23,24] Contraindications to blood salvage include infection and malignant disease. The risks of using autotransfusion are mainly related to blood quality and systemic effects on the patient. One example is high free haemoglobin level in salvaged blood due to haemolysis secondary to surgical trauma, suctioning techniques and vacuum aspiration.[25] The systemic levels of free haemoglobin after autotransfusion are however depending on several factors such as amount of retransfused blood, age, sex and medical status of the patient but the clinically critical plasma levels of free haemoglobin have not been determined. Another risk may be related to the potassium concentration in salvaged blood which is dependent on many factors such as degree of cell haemolysis and time elapsed before transfusion. Also, intra-operatively salvaged blood has a low concentration of fibrinogen and of factors V, VIII and X[26,27] and processing of blood leads to activation of complement and formation of pro-inflammatory cytokines.[28,29] Postoperatively shed blood has a high concentration of complement anaphylatoxins as well as IL-1, IL 6 and IL-8.[30,31] High levels of inflammatory mediators may cause the development of circulatory instability and pulmonary insufficiency but may also reduce immunosuppression after surgery and the risk of

postoperative infections.[32] In contrast to the possible risk of autotransfusion there are also possible benefits. E.g. the leukocyte count in salvaged blood is close to that of normal blood, salvaged red cells show normal morphology in contrast to allogeneic donor cells[33] and the concentration of 2.3-diphosphoglycerate is higher in salvaged blood than in banked blood which is associated with higher oxygen delivery capacity.[34] Today, a widespread experience with blood salvage and autotransfusion exists and the reported complications are few. A recommendation for a limit of 1500 ml in intra-operative use and of 2000 ml in postoperative use is considered to be safe.

Clinical efficacy of autologous blood transfusion by means of reduction in allogeneic blood transfusion has been confirmed in the literature for different type of systems[e.g. 6,14,40]
The Sangvia® Blood Salvage System, a filtered autotransfusion system developed by Astra Tech AB, has however not been specifically investigated with regards to efficacy. The product has been tested in pre-clinical and clinical studies and the quality of intra-operative salvaged blood[35-37] and the systemic effect after transfusion[38,39] has been investigated. The results indicate that the blood quality is similar to that of other filtrated system, e.g. postoperative blood salvage with Bellovac® ABT (Astra Tech AB) and that the system is safe to use in man.[38,39]

1.2 Rationale

Transfusion of postoperative autologous blood has been found to reduce the need for allogeneic blood transfusion[14, 40-42] and also reduce the number of postoperative infections.[43,44] Publications on the effect of intraoperative filtrated whole blood collection and transfusion are however today very limited or non-existing. The purpose of this study is to document the efficacy, measured by allogeneic blood transfusion rate, of the Sangvia® Blood Management System when used for intra-operative, and if bleeding continues after surgery, possibly also postoperative autologous whole blood transfusion in total hip replacement surgery. The study will also add safety data to previously reported studies.

2 STUDY OBJECTIVES

The overall study objective is to investigate efficacy, with regards to allogeneic blood transfusion rate, in total hip replacement patients receiving intra- and possibly also postoperative autologous blood with the Sangvia® Blood Management System compared to patients receiving no autologous transfusion.

2.1 Primary objective

The primary objective of this study is to compare clinical efficacy in total hip replacement patients receiving either blood transfusion with the Sangvia® Blood Management System or no autologous transfusion by assessment of need for allogeneic blood transfusion.

2.2 Secondary objectives

The secondary objectives of the study are….

….to observe and compare clinical efficacy by means of postoperative infection rates, use of postoperative antibiotics, length of hospital stay and health status for patients transfused with Sangvia® blood, no blood and/or allogeneic blood.

….to observe and compare clinical efficacy by means of systemic postoperative haemoglobin levels for patients transfused with Sangvia® blood, no blood or allogeneic blood.

….to collect details in health care utilisation (medications, in-patient service use, blood management) that would allow a calculation and comparison of cost-consequences applying either alternative in the blood management and from the perspective of the payer

….to observe and compare safety by means of adverse events, highlighting renal failures and events potentially related to transfusion, body temperature and other clinical complications for patients transfused with Sangvia® blood, no blood or allogeneic blood.

3 STUDY PLAN AND PROCEDURES

3.1 Overall study design and flow chart

This Clinical Study Protocol has been subjected to a peer review according to Astra Tech standard procedures.

The study is an assessor blinded, prospective, randomized, controlled, multi-centre investigation including a total of 300 patients scheduled for either primary or secondary total hip arthroplasty. The patients will be randomised to receive either intra-operative, and if bleeding continues after surgery, possibly also post-operative autologous blood transfusion with the Sangvia® system (test group) or no use of autologous blood, i.e. allogeneic blood transfusion when needed, (control group). For the test group, the autologous collected blood will be returned routinely for all patients. Exceptions are only allowed if the responsible investigator identifies an increased risk for the individual patient based on medical judgement. In the control group transfusion of allogeneic blood will be given based on the following transfusion triggers in combination with specific clinical considerations for each individual patient.

Transfusion triggers

Haemoglobin concentration	Clinical symptoms
≤ 8.5 g/dl<br>(≤5.3 mmol/l)*	-
> 8.5 g/dl<br>(>5.3 mmol/l)*	Signs of anemia as deemed by the investigator, e.g. tachycardia and/or hypotension etc.

*1 g/dl = 0.6206 mmol/l

Bank blood transfusion can be given to the test group as an addition to the autologous blood using the same transfusion triggers and clinical considerations as in the control group.
All bank blood transfusion will be based on transfusion triggers and determined by an assessor unaware of the treatment group whenever possible and the reason/s for the transfusion will be registered. For cases when the decision of bank blood transfusion are taken by a responsible investigator aware of the treatment allocation, e.g. in an emergency situation during surgery, the registered reasons for transfusion will be reviewed by an independent assessor/committee who are unaware of the treatment allocation. Inconsistency in transfusion decision between the

independent assessor/committee and the responsible investigator will be reflected in the per protocol analysis where patients with suspected biased transfusion decisions will be excluded.

To ensure equal distribution of primary and secondary total hip surgery in the test and control group patients will be stratified for this before randomisation, see Figure 1.

All patients will be followed during their hospital stay and at 2 months after discharge.

Screening and inclusion

- After obtaining the subject's written informed consent and verifying that the subject fulfils all inclusion and none of the exclusion criteria the subject will be included in the study and given a Subject Id.
- A reference venous blood sample/s will be taken from each subject before surgery, for analyses of Hb, haematocrit/erythrocute particle count (Hct/EVF), sodium (Na^+), potassium (K^+), thrombocyte particle count (TPC), C-reactive protein (CRP), leukocyte particle count (LPC), creatinine and Glomerular Filtration Rate (GFR)/Creatinine Clearance (C_{Cr}).

Haemoglobin (Hb) concentration > 9.0 g/dl (5.6 mmol/l) and normal creatinine/clearance levels will be verified.

- Subject ID will be allocated based on clinic number and procedure (primary or secondary surgery and treatment group given from randomization.
- Demographics will be collected and registered in the Case Report Form (CRF).
- Medical and surgical history as well as medications will be registered in the CRF.

Preoperative

- Before start of surgery, i.e. before general anaesthesia the Sangvia® system will be set up according to the user instructions, if applicable.
- The start time and procedures of surgery will be registered.
- Presence of SIRS will be evaluated.
- Vital signs (blood pressure, body temperature and heart rate) will be registered.
- Additional medications will be registered in the medication log.
- Clinical signs or symptoms of infection will be registered.

Post-operative follow-up

3 (±1) hours after end of surgery

- Surgery stop time will be registered in the CRF
- The estimated intra-operative and post-operative blood loss volumes will be registered in the blood loss log.
- Any allogeneic and/or autologous blood transfusions will be registered in the allogeneic and/or the autologous transfusion logs.
- The Sangvia® postoperative drain (test group) and the Bellovac drain (control group) connection time will be registered.
- Venous blood sample/s will be taken from each subject before return of post-operative autologous blood if applicable for analyses of Hb, Hct/EVF, Na^+, K^+, TPC, CRP, LPC, creatinine and Glomerular Filtration Rate (GFR)/Creatinine Clearance (C_{Cr}).
- For the patients randomized to the Sangvia® treatment group postoperative blood salvage will be stopped, returned and the kit disconnected within 6 hours after activation. The drain will remain connected until the first post-operative day. In total, the transfusion volume of 2500 ml must not be exceeded when combining the intra- and postoperatively collected autologous blood.
- Drainage will continue in both treatment groups.
- Any allogeneic and/or autologous blood transfusions will be registered in the allogeneic and/or the autologous transfusion logs.
- Presence of SIRS will be evaluated.

- Vital signs (blood pressure, body temperature and heart rate) will be registered.
- Any additional adverse events and/or medications will be registered in applicable logs.
- Clinical signs or symptoms of general and/or wound infection will be registered.

Day 1 - first post-operative morning, 24 (±6) hours after end of surgery
- Venous blood sample/s will be taken from each subject 24 hours after end of surgery for analyses of Hb, Hct/EVF, Na^+, K^+, TPC, CRP, LPC, creatinine and Glomerular Filtration Rate (GFR)/Creatinine Clearance (C_{Cr}).
- The estimated additional post-operative blood loss volume will be registered in the blood loss log, if applicable.
- Drainages in both treatment groups will be removed.
- Any additional allogeneic blood transfusions will be registered in the allogeneic transfusion log.
- Presence of SIRS will be evaluated
- Vital signs (blood pressure, body temperature and heart rate) will be registered.
- Any additional adverse events and/or medications will be registered in applicable logs.
- Clinical signs or symptoms of general and/or wound infection will be registered.

Day 2 – 48 (±6) hours after end of surgery
- Venous blood sample/s will be taken from each subject 48 hours after end of surgery for analyses of Hb, Hct/EVF, Na^+, K^+, TPC, CRP, LPC, creatinine and Glomerular Filtration Rate (GFR)/Creatinine Clearance (C_{Cr}).
- Any additional allogeneic blood transfusions will be registered in the allogeneic transfusion log.
- Vital signs (blood pressure, body temperature and heart rate) will be registered.
- Any additional adverse events and/or medications will be registered in applicable logs.
- Clinical signs or symptoms of general and/or wound infection will be registered.

Day 3 – 72 (±6) hours after end of surgery
- Any additional allogeneic blood transfusions will be registered in the allogeneic transfusion log.
- Vital signs (blood pressure, body temperature and heart rate) will be registered.
- Any additional adverse events and/or medications will be registered in applicable logs.
- Clinical signs or symptoms of general and/or wound infection will be registered.

Day 4 – 96 (±6) hours after end of surgery
- Venous blood sample/s will be taken from each subject 4 days after end of surgery for analyses of Hb, Hct/EVF, Na^+, K^+, TPC, CRP, LPC, creatinine and Glomerular Filtration Rate (GFR)/Creatinine Clearance (C_{Cr}).
- Any additional allogeneic blood transfusions will be registered in the allogeneic transfusion log.
- Vital signs (blood pressure, body temperature and heart rate) will be registered.
- Any additional adverse events and/or medications will be registered in applicable logs.
- Clinical signs or symptoms of general and/or wound infection will be registered.
- The patients will fill out a health status questionnaire.

Discharge
An assessment at discharge will be performed if discharge occurs later than 4 days after surgery.
- Any additional allogeneic blood transfusions will be registered in the alllogenic transfusion log.
- Vital signs (blood pressure, body temperature and heart rate) will be registered.
- Any additional adverse events and/or medications will be registered in applicable logs.
- Clinical signs or symptoms of general and/or wound infection will be registered.

Month 2 – 5-12 weeks after end of surgery

The patient will be seen at a post-op visit at approximately 2 months after discharge. If a follow-up visit is impossible a telephone call will be made instead.
- Any additional adverse events and/or medications will be registered in applicable logs.
- Clinical signs or symptoms of general and/or wound infection will be registered.
- The patients will fill out a health status questionnaire.

Figure 1 Study flow chart

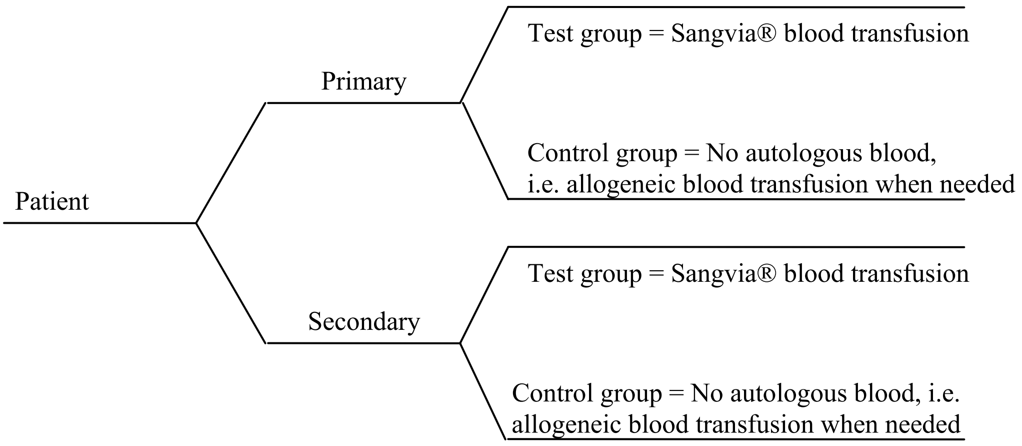

Table 1 Study plan

	Screening	Pre-op	Post-op follow up						
**Visit no/sampling time**	**1**	**2**	**3**	**4**	**5**	**6**	**7**	**8**	**9**
**Visit Description**	**Screening**	**Pre-op**	**3 h**	**Day 1**	**Day 2**	**Day 3**	**Day 4**	**Discharge**	**Month 2**
**Visit Window** (Hours/days ± No. Hours)	**Before or at admission**	**Before general anaesthesia**	**3h (± 1h), after end of surgery and before post-op transfusion if applicable**	**24h (± 6h) after end of surgery**	**48h (± 6h) after end of surgery**	**72h (± 6h) after end of surgery**	**96h (± 6h) after end of surgery**	**At day of discharge**	**8w (5-12w) after discharge**
Informed consent	X								
Medical history	X								
Inclusion/exclusion criteria	X								
Physical examination	X								
Demographics	X								
Blood loss log			X	X					
Drain management			X	X					
Hb and Hct/EVF	X		X	X	X		X		
TPC	X		X	X	X		X		
LPC	X		X	X	X		X		
Na^+, K^+	X		X	X	X		X		
Creatinine, GFR/C_{Cr}	X		X	X	X		X		
CRP	X		X	X	X		X		
SIRS		X	X	X					
Surgery information			X						

	Screening	Pre-op	Post-op follow up						
**Visit no/sampling time**	**1**	**2**	**3**	**4**	**5**	**6**	**7**	**8**	**9**
**Visit Description**	**Screening**	**Pre-op**	**3 h**	**Day 1**	**Day 2**	**Day 3**	**Day 4**	**Discharge**	**Month 2**
**Visit Window** (Hours/days ± No. Hours)	**Before or at admission**	**Before general anaesthesia**	**3h (± 1h), after end of surgery and before post-op transfusion if applicable**	**24h (± 6h) after end of surgery**	**48h (± 6h) after end of surgery**	**72h (± 6h) after end of surgery**	**96h (± 6h) after end of surgery**	**At day of discharge**	**8w (5-12w) after discharge**
Vital signs (blood pressure, body temperature and heart rate)	X	X	X	X	X	X	X	X	
Allogeneic transfusion log			X	X	X	X	X	X	
Adverse events, including clinical symptoms of infection			X	X	X	X	X	X	X
Medications	X	X	X	X	X	X	X	X	X
Wound infection			X	X	X	X	X	X	X
Health status questionnaire								X	X

Hb = Haemoglobin concentration (g/dl)
Hct/EVF = Haematocrit/Erythrocyte Volume Fraction (%)
Na^+ = Sodium (mmol/l)
K^+ = Potassium (mmol/l)
TPC = Thrombocyte Particle Concentration (10^9/l)
LPC = Leukocyte Particle Concentration (10^9/l) with applicable differentiation in percentage of neutrophils, eosinophils, basophils, lymphocytes, monocytes and macrophages.
CRP = C-reactive protein concentration (mg/l)
Creatinine = Serum creatinine (µmol/l), GFR = Glomerular Filtration Rate, C_{Cr} = Creatinine Clearance (ml/min/1.73m^2)
SIRS = systemic inflammatory response syndrome, i.e. two or more of the following

- Body temperature >38°C or <36°C
- Heart rate > 90 beats/minute
- Respiratory rate > 20 breaths/minute or $PaCP_2$ < 32mmHg
- LPC > $12x10^9$/l or < $4x10^9$/ml or 0.1% immature neutrophiles

3.2 Rationale and risk/benefit assessment

The study is designed as an assessor blinded, prospective, randomised and controlled efficacy study of use of intra-operative, and if bleeding continues after surgery, possibly also post-operative autologous blood salvage with the Sangvia® system.

Study population
Total hip replacements have been selected as study population for several reasons. Firstly, the majority of bleeding is considered to occur intra-operatively and thus enables collection and transfusion with the Sangvia® system. Secondly, today not all subjects subjected to total hip replacement receive allogeneic transfusion since the expected benefits are not considered to outweigh the risks and/or costs of it. Even so many of the subjects may benefit from transfusion depending on general medical condition and age. The risks of allocating subjects to a group where autologous blood collected with Sangvia® is transfused are considered low since previously findings and studies have verified safe use of the system. In addition, if allogeneic blood transfusion is needed it may be given in addition to the Sangvia® blood.

In the study both intra- and postoperative autologous blood collection and transfusion with the Sangvia® system is allowed in the test group since this is the intended use of the product and best reflects the clinical reality.

Primary variable
The choice of the primary variable for efficacy is based upon reported findings in the literature.[e.g.6,14,40,41] Need for allogeneic blood transfusion is considered to best reflect the blood loss and transfusion frequency in a patient population while other potential primary variables such as length of hospital stay and post-operative haemoglobin values are multi-factorial dependent on for example preferences of the clinic and gender of the patient etc.

The comparison between the treatment alternatives may be biased as it is almost impossible to blind the study. To mitigate the risk of bias the decision of bank blood transfusion will be taken based on transfusion triggers by an assessor unaware of the treatment group whenever possible and the reason/s for the transfusion will be registered. For cases when the decision of bank blood transfusion are taken by a responsible investigator aware of the treatment allocation, e.g. in an emergency situation during surgery, the registered reasons for transfusion will be reviewed by an independent assessor/committee who are unaware of the treatment allocation. Inconsistency in transfusion decision between the independent assessor/committee and the responsible investigator will be reflected in the per protocol analysis where patients with suspected biased transfusion decisions will be excluded. Also post-operative haemoglobin values will be monitored during the study period. In this way systematically inadequate and/or absent allogeneic blood transfusion decisions will be disclosed and addressed during monitoring visits and concerned patients can be excluded from the Per Protocol analysis.

Bleeding volume or complexity of surgery, i.e. surgery time, are considered to possibly influence outcome of the primary variable why stratification for either primary or secondary/revision hip surgery will be made at each recruiting clinic to ensure equal distribution in the two groups. Subgroup analyses of type (primary and secondary) and complexity (easy < 1 hour, medium 1-2 hours and complex > 2 hours) of surgery are planned to further investigate the correlation between the primary outcome and these factors. Age is also a factor that may have an impact on the outcome of the primary variable. It is however considered that randomization as such will ensure approximately equal age distribution in the two treatment groups. Differences in age

between the treatment groups will be evaluated and tested using Wilcoxon rank sum test in the analysis.

Comparator
The control group in the study consists of patients receiving allogeneic blood transfusion based on transfusion triggers and clinical judgement. This is considered as the golden standard since the majority of blood transfusions given in relation to major orthopaedic surgery are allogeneic. Drainages will be placed and used for 24 hours for both treatment groups to standardise post-operative routines and minimise differences between the two treatment groups.

3.2.1 Risk/benefit assessment

The risks for the subjects of using a filtered system for intra- and post-operative blood salvage are mainly related to uncontrolled blood quality. For the Sangvia® system these risks have been addressed by means of extended pre-clinical and clinical studies and extensive clinical experience of autologous blood transfusion in general. The results from studies and experiences indicate that the Sangvia® system is safe to use in man. Any unforeseeable risks of receiving autologous transfusion will be mitigated by monitoring the subjects during the transfusion period. Any signs of a serious clinical event will abort the transfusion. In addition to monitoring the transfusion period the subjects will be closely followed during their hospital stay and checked at 2 months after discharge. All adverse events and complications will be registered.

Allogeneic blood transfusion following major orthopedic surgery has been used for many years because of extensive blood loss leading to postoperative anemia and circulatory complications. The advantages are obvious, e.g. higher oxygen carrying capacity, reduced risk for cardiac, vascular and pulmonary complications etc. However, allogeneic transfusion is also associated with a number of potential risks, errors of matching, immediate and late immune reactions, immune suppression, increased rate of infection, absence of clotting factors and transmission of infectious diseases.

The possible benefits of the study are that the concept of reinfusion of intra- and post-operatively collected and filtered whole blood, in comparison to a group where allogeneic blood transfusion is the only transfusion option, will result in a reduced need for allogeneic blood transfusion and thus leading to lower costs and allogeneic blood related risks.

3.2.2 Ethical considerations

The study will be subjected to ethical review before any subjects are included.

The most important ethical consideration in the design of the current study is the comparative groups, i.e. that subject will be randomized to receive either autologous blood reinfusion or allogeneic blood transfusion when needed based on transfusion triggers. A randomized study of this kind may be considered as unethical but in order to get clinical evidence of treatment efficacy, a prospective, randomized study design is a prerequisite. Also, allogeneic blood transfusion is still considered as the golden standard and the long experience of using autologous blood indicate that this is a safe alternative.

Once a subject is enrolled, he or she has every right to withdraw the consent at any time during the course of the study without being subjected to any disadvantages. The decision to withdraw the consent will not be argued, even though a reason for the withdrawal may be asked for.

3.3 Selection of study population

3.3.1 Study selection record

Investigator(s) must keep a record of subjects who were considered for enrolment but were never enrolled e.g., subject screening log. This information is necessary to establish that the subject population was selected without bias.

3.3.2 Inclusion criteria

For inclusion in the study subjects must fulfill all of the following criteria:

1. Provision of informed consent.
2. Scheduled for primary or secondary, cemented or non-cemented, total hip arthroplasty.
3. Classified as ASA Physical Status Classification System class P1, P2 or P3 according to the American Society of Anaesthesiology.

3.3.3 Exclusion criteria

Any of the following is regarded as a criterion for exclusion from the study:

1. Involvement in the planning and conduct of the study (applies to both Astra Tech staff or staff at the study site).
2. Previous enrolment or randomisation of treatment in the present study.
3. Expected or confirmed participation in another clinical study, that may interfere with the present study, during the study period.
4. Suspected severe non-compliance to protocol as judged by the investigator.
5. Current symptoms of haemophilia.
6. Current symptoms of hyperkalaemia.
7. Current symptoms of systemic infection or local infection in the operation field.
8. Current symptoms of impaired renal function including creatinine/clearance levels above the normal reference values.
9. History of or presence of malignant disease with propensity for systemic spread during the last 5 years.
10. Current or expected use of cytotoxic drugs.

11. Current untreated anaemia (e.g. sickle cell anaemia), i.e. Hb concentration < 11 g/dl (7 mmol/l).

12. Use of recombinant erythopoeitin.

13. Use of aprotinin and/or fibrin sealant.

14. Use of other autologous blood transfusion than that with the Sangvia® system (e.g. CellSaver, pre-donation, acute normovolemic haemodilution etc.).

15. Women of childbearing age.

16. Fractures

17. Revision/secondary total hip surgery with expected serious bone grafting

3.3.4 Restrictions

Use of tranexamic acid (cyclokapron) is allowed as long as it is used for all included patients at the individual clinic.

3.3.5 Discontinuation of subjects from treatment or assessment

3.3.5.1 Criteria for discontinuation

Subjects may be discontinued from study treatment and study specific assessments at any time. Specific reasons for discontinuing a subject from this study are:

- Voluntary discontinuation by the subject who is at any time free to discontinue his/her participation in the study, without prejudice to further treatment
- Safety reasons as judged by the investigator and/or Astra Tech, e.g. new safety information.
- Severe non-compliance to protocol as judged by the investigator and/or Astra Tech
- Incorrect enrolment i.e., the subject does not meet the required inclusion/exclusion criteria for the study
- Subject lost to follow-up

3.3.5.2 Procedures for discontinuation

Subjects who discontinue should always be asked about the reason(s) for their discontinuation and the presence of any adverse events. If possible, they should be seen and assessed by an investigator. Adverse events shall be followed up.

3.3.5.3 Procedures for handling incorrect enrolled subjects

Subjects not meeting the inclusion/exclusion criteria for a study should, under no circumstances, be enrolled into the study - there can be no exceptions to this rule. Incorrect enrolled subjects will

not be included in the per protocol analysis set or in the efficacy conclusions and no financial compensation will be given for these subjects.

3.4 Treatments

3.4.1 Identity of investigational product and comparators

The investigational product is the CE-marked Sangvia® Blood Salvage System. The system consists of an intra-operative set, a post-operative set and vacuum tubing that enable collection, filtration and transfusion of intra-operative and post-operative blood.

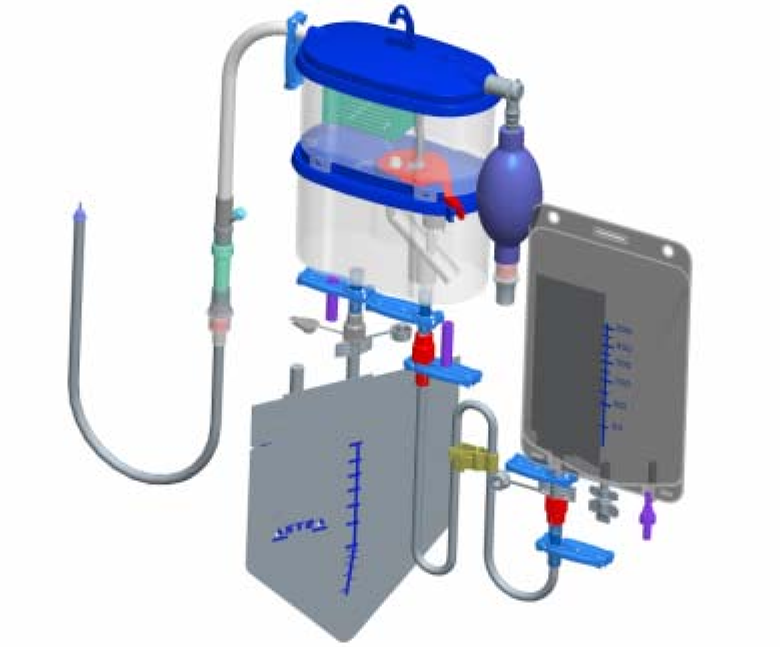

The recommended user instructions for the Sangvia® Blood Salvage System should be followed for set-up, blood collection and reinfusion.
In summary, the collection canister is connected to a suction source with the recommended suction pressure of 100 mmHg (maximum 150 mmHg is allowed) and a suction tip is connected to the inlet tubing of the system. The collection unit is placed lower or at the same height as the operation table and an anticoagulantia drip is connected to the system (ACD-A, sodium citrate or CPD) with the recommended ratio of 1:7 (approximately 20 drips per minute). The blood is filtered from larger debris, cement and bone particles by a macro filter of 200µm when collected in the chamber. The upper compartment of the collection unit has a maximum volume of 700 ml and the lower compartment has a maximum volume of 500 ml. When blood is being emptied to the transfusion bag the opening between the upper and the lower compartments should be closed. The blood is filtered from small particles by a micro filter before reinfusion. The reinfusion is initiated when 500 ml blood have been collected or at the end of surgery. Blood transfusion should be completed within 6 hours of the start of collection. Post-operative collection and transfusion will follow after surgery. The post-operative set is set-up and used according to the recommended user instructions. As for the intra-operative collection phase blood transfusion must be completed within 6 hours from start of collection during the post-operative phase. The reinfusion of the post-operatively collected blood may start either when the blood has passed 500 ml in the lower chamber or at the end of the 6 hour collection period. Not more than 2500 ml intra- and/or postoperatively collected blood in total should be transfused to the patients.

3.4.2 Treatment regimens

The subjects included in the study will be randomized to either receive blood transfusion with Sangvia® (test group) or to a group where only allogeneic blood transfusion is used if necessary (control group). In the test group the intra-, and if applicable also the post-operative blood, collected with Sangvia® will always be returned to the patient. In the control group allogeneic blood transfusion will be given if transfusion triggers have been fulfilled and/or if the clinical symptoms justify transfusion. Allogeneic blood transfusion is also allowed to be given in the test group in addition to the Sangvia® blood if transfusion triggers have been fulfilled and/or if the clinical symptoms justify transfusion. Every decision to give allogeneic blood transfusion must whenever possible be taken by an assessor, unaware of the treatment group, and based on his/her opinion of the complete clinical picture for the individual patient. In addition, the reason for a transfusion must always be registered in the CRF.

3.4.3 Labeling

The Sangvia® Blood Salvage System is a CE-marked system with a therefore applicable labeling. The labeling includes:

- "Sangvia® Blood Salvage System, intra-operative autologous blood reinfusion set"/ "Sangvia® Blood Salvage System, post-operative set"
- "Reference number"
- "Lot number"
- "Expiry date"
- "Sterile"
- "Single use"

The labeling will also include the study code (YA-DRA-0001), "Exclusively for clinical investigation" in local language and name and address of principal investigator and the sponsor.

3.4.4 Storage

All investigational products must be kept in a secure place to ensure exclusively use for this clinical study.

3.4.5 Accountability

Study products will not be distributed to the investigational site until all agreements between the investigator and Astra Tech are finalized and applicable ethical committee approvals have been obtained and communicated to Astra Tech. Distributed study products will be used only for this study and only in accordance with this Clinical Study Protocol. All product deliveries will be confirmed by the investigator or delegate. All unused study products must be returned to Astra Tech when treatment of the last subject has been completed. Any study product accidentally or deliberately destroyed must be accounted for and discrepancies between amounts dispensed and returned should be explained.

3.5 Method of assigning subjects to treatment groups

Each participating clinic will be given a range of Subject ID for use for their included patients. The distribution between clinics will be as follows:

Clinic No.	Subject ID range	Subject ID range for primary procedures	Subject ID range for secondary procedures
Clinic 1	1101-1299	1101-1199	1201-1299
Clinic 2	2101-2299	2101-2199	2201-2299
Clinic 3	3101-3299	3101-3199	3201-3299
Clinic 4	4101-4299	4101-4199	4201-4299
Clinic 5	5101-5299	5101-5199	5201-5299
Clinic 6	6101-6299	6101-6199	6201-6299

Subjects will be allocated their Subject ID in a strictly consecutive/sequential order when eligibility for inclusion has been established. Each Subject ID has a corresponding randomization code referring to the appropriate treatment allocation. The randomization process will be performed in a blinded approach, either with a web-based login system or by randomization envelopes that cannot be seen through without opening. If a subject discontinues from the study, the subject number will not be reused, and the subject will not be allowed to re-enter the study. However, during the recruitment period a drop-out subject may be replaced by a new included patient with the Subject ID next in turn in addition to the planned total number of subjects for the specific clinic (see section 6.1.3).

3.6 Blinding and procedures for unblinding the study

3.6.1 Methods for ensuring blinding

It is not possible to double blind this study due to the fact that it will be obvious for the study site team to which group the patient has been allocated. I.e. patients in the test group will have their blood collected with the Sangvia® device and in the control group no autologous blood salvage will be performed. The decision of blood bank transfusion will however be assessed in a blinded fashion with an independent assessors unaware of the patient treatment allocation. I.e. each decision of bank blood transfusion will be taken based on transfusion triggers by an assessor unaware of the treatment group whenever possible and the reason/s for the transfusion will be registered. For cases when the decision of bank blood transfusion are taken by a responsible investigator aware of the treatment allocation, e.g. in an emergency situation during surgery, the registered reasons for transfusion will be reviewed by an independent assessor/committee who are unaware of the treatment allocation. Inconsistency in transfusion decision between the independent assessor/committee and the responsible investigator will be reflected in the per protocol analysis where patients with suspected biased transfusion decisions will be excluded. Laboratory sample analyses will be done by assessors unaware of the patient treatment allocation.

3.6.2 Methods for unblinding the study

Not applicable.

3.7 Pre-study, concomitant and post-study treatment(s)

Subjects using cytotoxic drugs will be excluded from the study. Furthermore, the use of recombinant erythopoeitin for stimulation of the hematopoesis is not allowed during the study. Use of tranexamic acid (Cyclokapron) during surgery is allowed if all subjects at the individual clinic are treated in the same way. Other medication, which is considered necessary for the subject's safety and well-being, may be given at the discretion of the investigator(s). The administration of all medication must be recorded in the appropriate sections of the case report form (CRF).

3.8 Treatment compliance

To ensure treatment compliance the local study personnel must follow the recommended manuals and the instructions in this protocol for the investigational product.

4 MEASUREMENTS OF STUDY VARIABLES AND DEFINITIONS OF OUTCOME VARIABLES

4.1 Screening and demographic measurements

The following demographic data will be recorded via a standard Case Report Form (CRF):

- Date of birth
- Sex
- Significant medical and surgical history
- Medication at entry and during the study
- Type of anesthetics
- Surgical procedure and approach
- Weight and height (BMI)
- Systolic/diastolic blood pressure (mmHg)
- Body temperature (°C)
- Heart rate (beats per minute)
- Intra- and post-operative blood loss (ml);
  - Estimated by investigator
  - Uncompensated red cell blood loss will be calculated using the following formula
  Red blood cell loss (ml) = Estimated total body volume (ml)[52] x (Hct pre-op – Hct post-op)[53-55]

4.2 Primary outcome variable

4.2.1 Allogeneic blood transfusion

4.2.1.1 Methods of assessment

The need of allogeneic blood will be evaluated at 3 hours after surgery and at day 1, 2, 3, 4 after surgery and at discharge. Every transfusion of allogeneic blood during the study period will be registered in the allogeneic transfusion log.

4.2.1.2 Derivation or calculation of variable

Need for allogeneic blood transfusion will be expressed as transfusion rate in percentage, i.e. number of subjects with allogeneic blood transfusion/total number of subjects, and as the total volume amounts (in ml and expressed as units) of allogeneic blood transfused per treatment group. A transfusion index (units per transfused patient) will also be calculated.

4.3 Secondary outcome variable

4.3.1 Post-operative infection rate

Post-operative infection rate will be assessed by using clinical symptoms of infection, SIRS, specific evaluation of wound infection and assessment of CRP and LPC.

4.3.1.1 Methods of assessment

Clinical symptoms of infections:

Clinical symptoms of infections according to the classical signs of inflammation, i.e. swelling, redness, pain, heat and loss of function, and as deemed by the investigator will be regarded as an adverse event and registered in the AE log. Clinical symptoms of infection will be assessed at each check up after end of surgery throughout the study period.

SIRS:

Presence of systemic inflammatory response syndrome (SIRS)[45-47] will be evaluated pre-operatively and at 3 and 24 hours after end of surgery. The definition of SIRS is presence of two or more of the following observations

- Body temperature >38°C or <36°C
- Heart rate > 90 beats/minute
- Respiratory rate > 20 breaths/minute or $PaCP_2$ < 32 mmHg
- LPC > $12x10^9$/l or < $4x10^9$/ml or 0.1% immature neutrophiles

Wound infection:

Wound infections will be assessed at each check up after end of surgery throughout the study period. Ongoing wound infection will be classified as presence of:

- Purulent drainage or other sign of tissue/wound breakdown

and

- Increasing wound pain or tenderness[50,51]

CRP:

The C-reactive protein concentration given in mg/l will be assessed before surgery, at 3, 24 and 48 hours after surgery and at day 4 after surgery. Surgery induced inflammation and a CRP rise above 10 ml/l after 6 hours with a peak value not exceeding 150 mg/l at 48 hours. Postoperative infection will be defined as a maintained a raised CRP level after 48 hours or a secondary increase, i.e. values > 150 mg/l after 48 hours.

LPC:
The Leucocyte Particle Concentration given in count of 10^9/l will be assessed before surgery, at 3, 24 and 48 hours after surgery and at day 4 after surgery. A total count will be used as well as an applicable differentiation in percentage of neutrophils, eosinophils, basophils, lymphocytes (B-cells, T-cells, CD4+ (helper) T-cells, CD8+ (cytotoxic) T-cell and Natural Killer cells), monocytes and macrophages. Total LPC values of either higher than $12x10^9$/l or lower than $4x10^9$/ml will be considered as an indication of postoperative infection.

4.3.1.2 Derivation or calculation of variable

Each variable will be evaluated and presented separately and an overall post-operative infection rate will also be calculated.

4.3.2 Post-operative antibiotics use

4.3.2.1 Methods of assessment

The use of post-operative antibiotics will be assessed at each check up after surgery throughout the study period and registered in the MED log in the CRF.

4.3.2.2 Derivation or calculation of variable

Use of post-operative antibiotics will be expressed in percentage per treatment arm, i.e. number of subjects with post-operative antibiotics/total number of subjects. The total number of days and dose registered in the MED log for post-operative antibiotics will also be presented per treatment arm.

4.3.3 Length of hospital stay

4.3.3.1 Methods of assessment

The day of surgery will represent the start of the hospital stay, since the day of admission can, for various reasons, differ between each subject. The day of discharge will represent the last day of stay. The total length of stay will be divided on type of care facility, i.e. intensive care unit or post-operative care unit. Time per type of care facility will be registered in the CRF.

4.3.3.2 Derivation or calculation of variable

The days between surgery and discharge will be calculated and the total time and time per type of care facility will be compared between treatment groups for every clinic.

4.3.4 Post-operative Hb

4.3.4.1 Methods of assessment

Haemoglobin concentration in g/dl will be assessed together with haematocrit (erythrocyte volume fraction) in percentage pre-operatively and after 3 hours, 24 hours, 2 days and 4 days after surgery. Venous or capillary blood samples will be used.

4.3.4.2 Derivation or calculation of variable

Absolute differences between the treatment groups at each time point and in change from baseline (pre-operatively) will be evaluated.

4.4 Safety measurements and variables

The methods for collecting safety data are described below.

4.4.1 Adverse Events

4.4.1.1 Definitions

The definitions of Adverse Events (AEs), Adverse Device Effects (ADEs) and Serious Adverse Events (SAEs) are given below. It is of the utmost importance that all staff involved in the study are familiar with the content of this section. The principal investigator is responsible for ensuring this.

Adverse Event

An Adverse Event (AE) is any untoward and unintended medical occurrence in a subject. This definition does not imply that there is a relationship between the AE and the medical device under investigation. An AE which is possibly related, is one that may have been caused by the medical device, or treatment, however there is insufficient information to determine the likelihood of this possibility.

- Possibly related: temporal relationship of the onset of the event, relative to the use/administration of the medical device, is reasonable but the event could have been due to another, equally likely cause.
- Non-related (unlikely): temporal relationship of the onset of the event, relative to the use/administration of the medical device, is not reasonable or another cause can itself explain the occurrence of the event.

Ambiguous cases should be considered as possibly related.

Adverse Device Effect

An Adverse Device Effect (ADE) is any untoward and unintended response to a medical device. This definition includes any event resulting from insufficiencies or inadequacies in the instructions for use of the medical device or any event that is a result of a user error. This definition also includes treatment- or procedure- related events. ADEs can only occur from the time of medical device use/administration. Here the event is related to the use of the medical device where there is a probable/definite relationship that the event may have been caused by the medical device, or treatment.

- Probably related: temporal relationship of the onset of the event, relative to the use/administration of the medical device, is reasonable and the event is more likely explained by the medical device/treatment than by any other cause.

- Definitely related: temporal relationship of the onset of the event, relative to the use/administration of the medical device, is reasonable and there is no other cause to explain the event.

Serious Adverse Event

A Serious Adverse Event (SAE) is an AE/ADE occurring during any study phase of the medical device, that fulfils one or more of the following criteria:

- Results in death
- Is immediately life-threatening
- Requires in-subject hospitalisation or prolongation of existing hospitalisation
- Results in persistent or significant disability or incapacity
- Is a congenital abnormality or birth defect
- Is an important medical event that may jeopardise the subject or may require medical intervention to prevent one of the outcomes listed above.

If a non-related AE becomes serious, i.e. fulfilling one or more of the above criteria, action should be taken as for any other SAE.
For further guidance on the definition of a SAE and a guide to the interpretation of the causality question, see Appendix B to the Clinical Study Protocol.

4.4.1.2 Recording of Adverse Events and Adverse Device Effects

At each assessment point and/or visit, scheduled or unscheduled, the subject will be asked an open question; "Have you had any health problems since the previous visit?"

All AEs must be recorded in the CRF's AE log:
- Time of onset, action taken, outcome and whether the AE constitutes a SAE or not must be specified for events regarded as "possibly/probably/definitely" related to the device by the investigator.
- A short description and whether the AE constitutes a SAE or not must be specified for events regarded as non-related to the device by the investigator.

4.4.1.3 Reporting of Serious Adverse Events

Timelines for reporting of SAEs

Investigators and other study site personnel must inform appropriate Astra Tech representatives of any SAE that occurs during the course of the study within one day (i.e., immediately but no later than the end of the next business day) of when he or she becomes aware of it.
The Astra Tech representative will work with the Investigator to compile all the necessary information and ensure that the appropriate Astra Tech personnel receives a report by day one for all fatal and life-threatening cases and by day five for all other SAEs.
If an AE or ADE becomes a SAE, this and other relevant follow-up information must also be provided to Astra Tech within one day for all fatal and life-threatening cases and by day five for all other SAEs.
The Investigator is responsible for informing the ethics committee of SAEs as per local requirements.
Astra Tech is responsible for informing the regulatory authority of SAEs as per local requirements.

4.5 Patient-Reported Outcomes (PROs)

4.5.1 Health status questionnaire

4.5.1.1 Methods of assessment

Health status will be measured by means of a validated self reported quality of life questionnaire, i.e. the EQ-5D. The questionnaire will be completed 4 days after surgery and at the check up at 2 months.

4.5.1.2 Derivation or calculation of variable

Applicable scores and results from the questionnaire will be analyzed and presented according to user instructions from the provider. The results from the two treatment groups at day 4 and month 2 as well as the change between the two assessments will be compared.

4.6 Health Economic measurements and variables

4.6.1 Health care utilisation

4.6.1.1 Methods of assessment

Health care utilisation will be measured by length of hospital stay, type of care facility during hospital stay, use of medications, bank blood and the Sangvia® treatment.

4.6.1.2 Derivation or calculation of variable

Values for length of hospital stay, type of care facility, use of medications, bank blood and the Sangvia® treatment will be derived from the results from the current study. Unit costs will not be applied within the study.

4.7 Volume of blood sampling and handling of biological samples

The total volume of blood that will be drawn from each subject in this study is as follows:

Table 2 Volume of blood to be drawn from each subject

Sampling time/Visit No.	Sample volume (mL)	No. of samples	Total volume (mL)
1. Screening/Pre-op	5 ml	2	10 ml
4. 3 hours after end of surgery	5 ml	2	10 ml
5. 24 hours after end of surgery	5 ml	2	10 ml
6. 2 days after surgery	5 ml	2	10 ml
8. 4 days after surgery	5 ml	2	10 ml
**Total**		10	50 ml

4.7.1 Analysis of biological samples

4.7.1.1 Clinical chemistry samples

Blood markers related to routine safety monitoring after surgery will be analysed, i.e. haemoglobin concentration (g/dl), haematocrit/Erythrocyte Volume Fraction (%), sodium (mmol/l), potassium (mmol/l), Thrombocyte Particle Concentration (10^9/l), Creatinine (µmol/l) and Glomerular Filtration Rate (GFR)/Creatinine Clearance (C_{Cr}) (ml/min/1.73m^2).

Analyses will be made according to the schedule below:

	Screening/ Pre-op (1)	3 hours post-op (4)	24 hours post-op (5)	2 days post-op (6)	4 days post-op (8)
**Hb**	X	X	X	X	X
**Hct/EVF**	X	X	X	X	X
**TPC**	X	X	X	X	X
**LPC**	X	X	X	X	X
**Na**	X	X	X	X	X
**K**	X	X	X	X	X
**Creatinine, GFR/ C_{Cr}**	X	X	X	X	X
**CRP**	X	X	X	X	X

Hb, Hct/EVF, TPC, LPC in one EDTA container, 5 ml blood..
Na^+, K^+, CRP and creatinine should be taken in one gel container (e.g. Hettich Lab Instruments), 5 ml blood.

5 DATA MANAGEMENT

Astra Tech will coordinate Data Management activities.

Clinical Subject data will be collected via paper CRFs, and the collected data will be entered into a Clinical Data Management System called Oracle Clinical. Data will be recorded in the pCRFs only by authorized study site personnel at the investigational sites.

The study site personnel will be responsible for recording data on specified observations, tests and assessments specified in the protocol. Data editing for correction or clarification purposes will be made before the pCRFs are collected by the monitor and submitted for data entry at Astra Tech.
Data will be continuously entered and validated in Oracle Clinical at Astra Tech during the study. Should questionable data be detected during the validation process, written queries will be raised as result of this validation. The study site personnel are required to resolve any such queries.

At the end of the study, Astra Tech will perform final validation checks, including central consistency checks, after which Clean File will be declared and the database locked.

The study Data Management Plan will describe in greater detail the methods used to collect, check, and process clinical data. It will also clarify the roles and responsibilities of the various functions and personnel involved in the data management process.

6 STATISTICAL METHODS AND DETERMINATION OF SAMPLE SIZE

6.1 Statistical evaluation – general aspects

When using the terminology descriptive statistics it is meant that the number of subjects, mean, median, standard deviation, minimum and maximum values will be presented for continuous data and frequencies and percentages for categorical data. If nothing else is stated, descriptive statistics will be given for each variable in the study.

A p-value below 5% is called statistically significant even though it is recognized that multiple secondary hypotheses will be tested.

A comprehensive Statistical Analysis Plan (SAP) may be prepared before database lock. If prepared, the Statistical Analysis Plan will be kept as an appendix to the Data Management Plan.

6.1.1 Demographics and other baseline characteristics

Demographics and other baseline characteristics will be presented by means of descriptive statistics.

6.1.2 Covariates and prognostic variables

Bleeding volume or complexity of surgery, i.e. surgery time, are considered to possibly influence outcome of the primary variable why stratification for either primary or secondary/revision hip surgery will be made at each recruiting clinic to ensure equal distribution in the two groups. Subgroup analyses of type (primary and secondary) and complexity (easy < 1 hour, medium 1-2 hours and complex > 2 hours) of surgery are planned to further investigate the correlation between the primary outcome and these factors.
Age is also an potential factor that may have an impact on the outcome of the primary variable. It is however considered that randomization as such will ensure approximately equal age distribution in the two treatment groups. Differences in age between the treatment groups will be evaluated and tested using Wilcoxon rank sum test in the analysis.

6.1.3 Handling of dropouts and missing data

Subjects dropping out from the trial before surgery will be replaced. Subjects dropping out after surgery during the recruitment period will be compensated for using continuous recruitment. Subjects dropping out after surgery and after the completion of the recruitment period will be compensated for in the sample size estimation, see section 6.5.

6.1.4 Multi-centre

This study is a multi-centre study and there is no a priori reason to suspect that there will be any qualitative differences between the centres. However, local treatment routines at different clinics

are considered to potentially affect the outcome of the primary variable why stratification for each clinic will be made. This is considered to ensure equal distribution between test and control group patients why primary statistical analysis will not include centre in the model. The result of the primary variable will however be presented by centre as part in subgroup analyses.

6.2 Description of outcome variables in relation to objectives and hypotheses

The primary variable, transfusion rate, will be measured as the proportion (π) of patients in each treatment group that is receiving allogeneic blood transfusion of the total number of patients in each treatment group. The hypothesis to be tested is:

$H_0 = \pi_s = \pi_c$
versus
$H_1 = \pi_s \neq \pi_c$
Where:
π_S = Proportion patients treated with Sangvia® that require allogeneic blood transfusion in addition to Sangvia® transfusion.
π_c = Proportion patients in the control group that require allogeneic blood transfusion.

The hypothesis will be tested using the Fischer Exact Test. The hypothesis will be rejected if the p-value is ≤ 0.05.

Secondary objectives will be tested using similar hypotheses. The total and variable specific proportion values for infection rate, use of post-operative antibiotics and adverse events will be tested between the groups using the Fischer Exact Test. Continuous variables (e.g. post-op Hb values) will be compared and tested using the Wilcoxon Rank Sum test. Laboratory blood markers will also be evaluated for changes over time within each treatment group and this change will be tested using the Wilcoxon Signed Rank test.

6.3 Description of analysis sets

The study will be analyzed using a Per Protocol (PP) and an All Patient Treated (APT) approach. Subjects fulfilling the inclusion criteria but none of the exclusion criteria and have been treated according to the protocol without any major deviations (e.g. wrongful transfusion decision of bank blood) will be included in the PP analysis set. All patients will be included in the APT analysis set. Treatment efficacy related conclusions will be based on the results from the PP analysis and demography and treatment safety related conclusions will be based on the results from the APT analysis.

Subgroup analyses of type (primary and secondary) and complexity (easy < 1 hour, medium 1-2 hours and complex > 2 hours) of surgery will be performed as well as analyses per clinic and per blood transfusion type (none, autologous, allogeneic and combinations).

6.4 Method of statistical analysis

Fischer Exact test will be used to test frequencies of dichotomous response variables. The non-parametric Wilcoxon Rank Sum test will be used to test differences in continuous response

variables between the parallel treatment groups. Wilcoxon Signed Rank test will be used to test differences over time within each treatment group. P-values equal to or below 0.05 are considered as statistically significant.

6.5 Determination of sample size

The primary variable and objective is the base for the sample size calculation. The sample size formula used is referred to in Lachin 1981.[48]

$$N = \frac{\left(Z_\alpha + Z_\beta\right)^2 4\bar{\pi}(1-\bar{\pi})}{(\pi_S - \pi_c)^2}$$

Where:

N = total number of patients
Z_α = 1.96 (α= 0.05)
Z_β = 1.2816 (1-β = 0.90)

$$\bar{\pi} = \frac{\pi_s + \pi_c}{2}$$

An estimation normal use of allogeneic blood of 21% is based on reported values in the literature.[14,40,42,49] It is acknowledged that in revision surgery a higher percentage of allogenic blood transfusions is seen than in primary total hip replacements. Because revision patients requiring serious bone grafting are excluded, the percentage of revision patients is expected to be just slightly higher than for the average primary total hip. A high number of revision, e.g. a maximum of 30%, in the total group of patients is considered to have limited effect of the total transfusion rate but are accounted for in the 21% assumed for the total group.The expected values of transfusion rate in the Sangvia® group is estimated to lie around 7%. Accordingly, imputing the values for the estimated transfusion rates in the test and control group generates a mean transfusion rate value of 14%. The sample size necessary for detection of this expected difference in transfusion rate frequency is calculated to 260 patients (5% significance level, two-sided hypothesis, 90% power). Additional 40 patients will be included in the study to compensate for drop-out. In summary, 300 patients will be included in total and 150 in each treatment group.

The transfusion rate of bank blood is considered to vary dependent on type and complexity of surgery. Greatest normal use and differences between the groups are expected in the subgroup with medium (surgery duration 1-2 hours) to complex (surgery duration more than 2 hours) surgery complexity why power are considered to be adequate for the planned subgroup analyses in these groups.

6.6 Statistical analyses during the course of the study

A conditional sample size estimation is performed why estimated values of allogeneic transfusion rate in the test and control group will be confirmed in an interim analysis when 150 patients have completed the study. If the normal transfusion rate of allogeneic blood is lower than 21% or the

transfusion rate in the test group is higher than 7% the study risk being inconclusive. In such cases additional patients may be considered after approval from the IEC.

7 STUDY MANAGEMENT

7.1 Monitoring

Before first subject enters into the study, a representative of Astra Tech will visit the investigational study site to:

- Determine the adequacy of the facilities
- Discuss with the investigator(s) (and other personnel involved with the study) their responsibilities with regard to protocol adherence, and the responsibilities of Astra Tech or its representatives. This will be documented in a Clinical Study Agreement between Astra Tech and the investigator

During the study, a monitor from Astra Tech or company representing Astra Tech will have regular contacts with the study site, including visits to:

- Provide information and support to the investigator(s)
- Confirm that facilities remain acceptable
- Confirm that the investigational team is adhering to the protocol, that data are being accurately recorded in the CRFs, and that investigational product accountability checks are being performed
- Perform source data verification (a comparison of the data in the CRFs with the subject's medical records at the hospital or practice, and other records relevant to the study). This will require direct access to all original records for each subject (e.g., clinic charts).

The monitor or another Astra Tech representative will be available between visits if the investigator(s) or other staff at the centre need information and advice.

7.2 Audits and inspections

Authorized representatives of Astra Tech, a regulatory authority, an Ethics Committee may visit the centre to perform audits or inspections, including source data verification. The purpose of an Astra Tech audit or inspection is to systematically and independently examine all study-related activities and documents to determine whether these activities were conducted, and data were recorded, analyzed, and accurately reported according to the protocol, ISO 14155, Good Clinical Practice (GCP) and any applicable regulatory requirements. The investigator should contact Astra Tech immediately if contacted by a regulatory agency about an inspection at his or her centre.

7.3 Training of staff

The principal investigator will maintain a record of all individuals involved in the study (medical, nursing and other staff). He or she will ensure that appropriate training relevant to the study is given to all of these staff, and that any new information of relevance to the performance of this study is forwarded to the staff involved.

7.4 Changes to the protocol

Study procedures will not be changed without the mutual agreement of the principal investigators and Astra Tech. If it is necessary for the study protocol to be amended, the amendment and/or a new version of the study protocol (Amended Protocol) must be notified to or approved by each Ethics Committee, and if applicable, also the local regulatory authority, before implementation. Local requirements must be followed. If a protocol amendment requires a change to a particular centre's Informed Consent Form, then Astra Tech and the centre's Ethics Committee must be notified. Approval of the revised Informed Consent Form by Astra Tech and by the Ethics Committee is required before the revised form is used.
Astra Tech will distribute administrative changes, amendments and new versions of the protocol to each principal investigator.

7.5 Study agreements

The principal investigator at each centre must comply with all the terms, conditions, and obligations of the Clinical Study Agreement for this study. In the event of any inconsistency between this Clinical Study Protocol and the Clinical Study Agreement, the Clinical Study Protocol shall prevail. In the event of any inconsistency between translations of the Clinical Study Protocol and/or the Clinical Study Agreement, the original English versions of the Clinical Study Protocol and the Clinical Study Agreement shall prevail.

7.6 Study timetable

Before a subject's enrolment in the study and any study-related procedures are undertaken the following should be fulfilled:

- Signed Clinical Study Protocol and other agreements between Astra Tech and the Principal Investigator/Study Site.

- Approval of the study by the Ethics Committee

Date of first subject enrolled is estimated to 28th February 2009 and date of last subject completed is estimated to the 30th November 2009.

8 ETHICS

8.1 Ethics review

Astra Tech will provide Ethics Committees and Principal Investigators with safety updates/reports according to local requirements.

The final study protocol, including the final version of the Informed Consent Form, must be approved or given a favorable opinion in writing by an Ethics Committee as appropriate. The investigator must submit written approval to Astra Tech before he or she can enroll any subject into the study.

The Principal Investigator is responsible for informing the Ethics Committee of any amendment to the protocol in accordance with local requirements. In addition, the Ethics Committee must approve all advertising used to recruit subjects for the study.

The Principal Investigator is also responsible for providing the Ethics Committee with reports of any SAEs (or ADEs) from any other study conducted with the investigational product. Astra Tech will provide this information to the Principal Investigator.

Progress reports and notifications of SAEs (and ADEs) will be provided to the Ethics Committee according to local regulations and guidelines.

8.2 Ethical conduct of the study

The study will be performed in accordance with ethical principles that have their origin in the Declaration of Helsinki and are consistent with ISO 14155 and Good Clinical Practice and applicable regulatory requirements.

8.3 Informed consent

The principal investigator(s) at each centre will ensure that the subject is given full and adequate oral and written information about the nature, purpose, possible risk and benefit of the study. Subjects must also be notified that they are free to discontinue from the study at any time. The subject should be given the opportunity to ask questions and allowed time to consider the information provided.
The subject’s signed and dated informed consent must be obtained before conducting any procedure specifically for the study, including the following:
The principal investigator(s) must store the original, signed Informed Consent Form in the ISF.
A copy of the signed Informed Consent Form must be given to the subject.
If modifications are made according to local requirements, the local version has to be approved by Astra Tech.

8.4 Subject data protection

The Master Informed Consent Form will incorporate (or, in some cases, be accompanied by a separate document incorporating) wording that complies with relevant data protection and privacy legislation. Pursuant to this wording, subjects will authorize the collection, use and disclosure of their study data by the Investigator and by those persons who need that information for the purposes of the study.
The Master Informed Consent Form will explain that study data will be stored in a computer database, maintaining confidentiality in accordance with national data legislation. All data computer processed by Astra Tech will be identified by a study code and a Subject ID.
The Master Informed Consent Form will also explain that for data verification purposes, authorized representatives of Astra Tech, a regulatory authority, an Ethics Committee may require direct access to parts of the hospital or practice records relevant to the study, including subjects' medical history.

9 PROCEDURES IN CASE OF EMERGENCY

9.1 Medical emergency

In the case of a medical emergency you may contact the Clinical Study Team Leader. If the Clinical Study Team Leader is not available, contact the Therapeutic Area Manager at Astra Tech, see below:

**Role in the study**	**Name**	**Address & telephone number**
Clinical Study Team Leader responsible for the protocol	Maria Åberg	Astra Tech AB<br>Att: Maria Åberg<br>Aminogatan 1, P.O. Box 14, SE-431 21 Mölndal, Sweden<br>+46 31 356 8274
Therapeutic Area Manager responsible for the protocol	Anna-Karin Sundin	Astra Tech AB<br>Att: Anna-Karin Sundin<br>Aminogatan 1, P.O. Box 14, SE-431 21 Mölndal, Sweden<br>+46 31 776 3292

The principal investigator(s) is responsible for ensuring that procedures and expertise are available to handle medical emergencies during the study. **A medical emergency usually constitutes an SAE and should be reported as such, see Section 4.4.1.1.**

9.2 Pregnancy

Pregnancy itself is not regarded as an AE/ADE unless there is a suspicion that the medical device under study may have interfered with the effectiveness of a contraceptive medication.

Congenital abnormalities/birth defects and spontaneous miscarriages should be reported as SAEs. Elective abortions without complications should not be handled as AEs/ADEs.

10 REFERENCES

1. Gilstad CW. Anaphylactic transfusion reactions. Curr Opin Hematol. 2003 Nov;10(6):419-23
2. Bux J. Transfusion-related acute lung injury (TRALI): a serious adverse event of blood transfusion. Vox Sang. 2005 Jul;89(1):1-10
3. Blajchman MA. Immunomodulation and blood transfusion. Am J Ther. 2002 Sep-Oct;9(5):389-95
4. Rees JE, Jeavons R, Dixon JH. An economic justification for autologous blood re-infusion in primary total knee replacement surgery. Ann R Coll Surg Engl. 2005 Mar;87(2):102-5
5. Waters JH. Indications and contraindications of cell salvage. Transfusion 2004;44(12 Suppl):40S-44S
6. Carless P, Moxey A, O'Connell D, Henry D. Autologous transfusion techniques: a systematic review of their efficacy. Transfus Med 2004;14(2):123-144
7. Rubens FD, Boodhwani M, Lavalee G, Mesana T. Perioperative red blood cell salvage. Can J Anaesth 2003;50(6):S31-S40
8. Monk TG. Acute normovolemic hemodilution. Anesthesiol Clin North America. 2005 Jun;23(2):271-81
9. Rock G, Bormanis J, Neurath D. The development of an optimized autologous blood donation program. Transfus Apher Sci. 2005 Nov;33(3):325-31.
10. Shiga T, Wajima Z, Inoue T, Sakamoto A. Aprotinin in major orthopedic surgery: a systematic review of randomized controlled trials. Anesth Analg 2005;101(6):1602-1607.
11. Dixon S, James V, Hind D, Currie CJ. Economic analysis of the implementation of autologous transfusion technologies throughout England. Int J Technol Assess Health Care. 2005 Spring;21(2):234-9
12. Bengtsson A, Bengtson JP. Autologous blood transfusion: preoperative blood collection and blood salvage techniques. Acta Anaesthesiol Scand. 1996 Sep;40(8 Pt 2):1041-56
13. Riddler BMF, Thompson JF. The Qualities of Blood Reinfused During Cell Salvage. TATM 2003;5(5):466-471
14. Strümper D, Weber EW, Gielen-Wijffels S, et al. Clinical efficacy of postoperative autologous transfusion of filtered shed blood in hip and knee arthroplasty. Transfusion 2004;44(11):1567-1571
15. Munoz M, Cobos A, Campos A, Ariza D, Munoz E, Gomez A. Impact of postoperative shed blood transfusion, with or without leucocyte reduction, on acute-phase response to surgery for total knee replacement. Acta Anaesthesiol Scand. 2005 Sep;49(8):1182-90.
16. Sinardi D, Marino A, Chillemi S, Irrera M, Labruto G, Mondello E. Composition of the blood sampled from surgical drainage after joint arthroplasty: quality of return. Transfusion. 2005 Feb;45(2):202-7
17. Friederichs MG, Mariani EM, Bourne MH. Perioperative blood salvage as an alternative to predonating blood for primary total knee and hip arthroplasty. J Arthroplasty. 2002 Apr;17(3):298-303
18. Daly KJ, Torella F, Ashleigh R, McCollum CN. Screening, diagnosis and advances in aortic aneurysm surgery. Gerontology. 2004 Nov-Dec;50(6):349-59
19. Bartels C, Bechtel JV, Winkler C, Horsch S: Intraoperative autotransfusion in aortic surgery: Comparison of whole blood autotransfusion versus cell separation. J Vasc Surg. 1996 Jul;24(1):102-8.
20. Jabbour N, Gagandeep S, Mateo R, Sher L, Genyk Y, Selby R. Transfusion free surgery: single institution experience of 27 consecutive liver transplants in Jehovah's Witnesses. J Am Coll Surg. 2005 Sep;201(3):412-7

21. Hughes LG, Thomas DW, Wareham K, Jones JE, John A, Rees M. Intra-operative blood salvage in abdominal trauma: a review of 5 years' experience. Anaesthesia. 2001 Mar;56(3):217-20
22. Ozmen V, McSwain NE Jr, Nichols RL, Smith J, Flint LM. Autotransfusion of potentially culture-positive blood (CPB) in abdominal trauma: preliminary data from a prospective study. J Trauma. 1992 Jan;32(1):36-9
23. Westerberg M, Bengtsson A, Jeppsson A. Coronary surgery without cardiotomy suction and autotransfusion reduces the postoperative systemic inflammatory response. Ann Thorac Surg. 2004 Jul;78(1):54-9
24. Schmidt H, Bendtzen K & Mortensen PE: The inflammatory response after autotransfusion of shed mediastinal blood. Acta Anaesthesiol Scand 42 : 558, 1998
25. Riou B, Arock M, Guerrero M, Ramos M, Thoreux P, Guillosson JJ, Roy-Camille R, Viars Haematological effects of postoperative autotransfusion in spinal surgery. Acta Anaesthesiol Scand. 1994 May;38(4):336-41.P
26. Krohn CD, Reikerås O, Bjørnsen S, Brosstad F (#2): Fibrinogen, fibrin and its degradation products in drained blood after major orthopaedic surgery. Blood Coagulation Fibrinolysis 1999 Jun;10(4):167-71.
27. Duchow J, Ames M, Hess T, Seyfert U: Activation of plasma coagulation for unwashed drainage blood after hip joint arthroplasty. J Arthroplasty. 2001 Oct;16(7):844-9.
28. Kristiansson M, Soop M, Saraste L, Sundqvist KG, Suontaka AM & Blombäck M: Cytokine and coagulation characteristics of retrieved blood after arthroplasty. Intensive Care Med. 1995 Dec;21(12):989-95.
29. Bottner F, Sheth N, Chimento GF, Sculco TP. Cytokine levels after transfusion of washed wound drainage in total knee arthroplasty: a randomized trial comparing autologous blood and washed wound drainage. J Knee Surg. 2003 Apr;16(2):93-7.
30. Dalen T, Bengtsson A, Brorsson B, Engstrom KG. Inflammatory mediators in autotransfusion drain blood after knee arthroplasty, with and without leucocyte reduction. Vox Sang. 2003 Jul;85(1):31-9.
31. Andersson I, Tylman M, Bengtson JP, Bengtsson A. Complement split products and pro-inflammatory cytokines in salvaged blood after hip and knee arthroplasty. Can J Anaesth. 2001 Mar;48(3):251-5.
32. Gharehbaghian A, Haque KM, Truman C, Evans R, Morse R, Newman J, Bannister G, Rogers C, Bradley BA. Effect of autologous salvaged blood on postoperative natural killer cell precursor frequency. Lancet. 2004 Mar 27;363(9414):1025-30
33. Kent P, Ashley S, Thorley PJ, Shaw A, Parkin A, Kester RC. 24-hour survival of autotransfused red cells in elective aortic surgery: a comparison of two intraoperative autotransfusion systems. Br J Surg. 1991 Dec;78(12):1473-5.
34. McShane AJ, Power C, Jackson JF, Murphy DF, MacDonald A, Moriarty DC, Otridge BW. Autotransfusion: quality of blood prepared with a red cell processing device. Br J Anaesth. 1987 Aug;59(8):1035-9
35. Astra Tech Internal Document: Complement split products and pro-inflammatory cytokines in salvaged blood during hip arthroplasty – a pilot study on Sangvia®. Clinical Study Report YA-DRA-0003-I. 28th August 2006
36. Astra Tech Internal Document: Influence of heparin coating on haemolysis, complement split products and pro-inflammatory cytokines in salvaged blood during hip arthroplasty – a pilot study on Sangvia®™. Clinical Study Report YA-DRA-0003-II. 1st August 2007
37. Kvarnström A, Schmidt A, Tylman M, Jacobsson M and Bengtsson A. Complement split products and proinflammatory cytokines in intraoperatively salvaged unwashed blood during hip replacement: comparison between heparin-coated and non-heparin-coated autotransfusion systems. Vox Sang. 2008;28

38. Astra Tech Internal Document: An open prospective study on intraoperative autologous transfusion of filtered whole blood with the Sangvia® Blood Salvage System. Clinical Study Report YA-DRA-0005. 17th December 2007
39. Pomianowski S, Michalik D, Król R, Stachura A and Bengtsson A. Intraoperative collection and transfusion of autologous whole blood - Is it safe? Transfusion Alternatives in Transfusion Medicines 2008;10 (Suppl 1):37
40. Moonen AFCM, Knoors NT, van Os JJ, Verburg AD and Pilot P. Retransfusion of filtered shed blood in primary total hip and knee arthroplasty: a prospective randomized clinical trial. Transfusion 2007;47:379-384
41. Newman JH, Bowers M and Murphy J. The clinical advantages of autologous transfusion. J Bone joint Surg [Br] 1997;79-B:630-2
42. Grosvenor D, Goyal V and Goodman S. Efficacy of postoperative blood salvage following hip arthroplasty in patients with and without deposited autologous units. J Bone Joint Surg Am 2000;82-A(7):951-4
43. Innerhofer P, Klingler A, Klimmer C, Fries D and Nussbaumer W. Risk for postoperative infection after transfusion of white blood clee-filtered allogeneic or autologous blood components in orthopedic patients undergoing primary arthroplasty. Transfusion 2005;45:103-110
44. Rosencher N, Kerkkamp HEM, Macheras G, Munuera LM, Menichella G, Barton DM, Cremers S and Abraham IL. Orthopedic surgery transfusion haemoglobin European overview (OSTHEO) study: blood management in elective knee and hip arthroplasty in Europe. Transfusion 2003;43:459-469
45. Farrer A, Spark JI, and Scott DJA. Autologous blood transfusion: The benefits to the patient undergoing abdominal aortic aneurysm repair. J Vasc Nurs 1997;15:111-5
46. Sarbinowski R, Arvidsson S, Tylman M, Öresland T and Bengtsson A. Plasma concentration of procalcitonin and systemic inflammatory response syndrome after colorectal surgery. Acta Anaesthesiol Scand 2005;49:191-196
47. Bone RC, Balk RA, Cerra FB, Dellinger RP, Fein AM, Knaus WA, Schein RM and Sibbald WJ. Definitions for sepsis and organ failure and guidelines for the use of innovative therapies in sepsis. The ACCP/SCCM Consensus Conference Committee. American College of Chest Physicians/Society of Critical Care Medicine. Chest 1992;101:1644-1655
48. Lachin JM. Introduction to sample size determination and power analysis for clinical trials. Controlled clinical trials 1981;2:93-113
49. Weber EWG, Slappendel R, Prins M, van der Schaaf DB, Durieux ME and Strümper D. Perioperative blood transfusions and delayed wound healing after hip replacement surgery: Effects on duration of hospitalisation. Anesth Analg 2005;100:1416-21
50. Bruce J, Russel EM, Mollison J, Krukowski ZH. The quality of measurement of surgical wound infection as the basis for monitoring: A systematic review. J Hosp Infection 2001;49:99-108
51. Gardner SE, Frantz RA, Doebbeling BN. The validity of the clinical signs and symptoms used to identify localized chronic wound infection. Wound Repair Regen 2001;9:178-86.
52. Nadler SB, Hidalgo JU, Bloch T. Prediction of blood volume in normal human adults. Surgery 1962;51:224-232
53. Brecher ME, Monk T, Goodnough LT. A standardized method for calculating blood loss. Transfusion 1997;37:1070-4
54. Hurle R, Poma R, Maffezzini M, Manzetti A, Piccinelli A, Taverna G, Bellavita P and Graziotti P. A simple mathematical approach to calculate blood loss in radical prostatectomy. Urologia Internationalis 2004;72:135-139
55. Monte del Trujillo M, Carrero A and Muños M. The utility of the perioperative autologous transfusion system OrthoPat® in total hip replacement surgery: a prospective study. Arch Orthop Traum Surg 2008;128:1031-1038

CLINICAL STUDY PROTOCOL: APPENDIX A

Study Product:	Sangvia®
Study Code:	YA-DRA-0001
Edition No.:	2
Date:	12th January 2009

Appendix A
Signatures

ASTRA
ASTRA TECH

Astra Tech Signatures

A prospective, randomized, controlled trial of retransfusion of intra-operatively collected filtered whole blood in total hip surgery

I agree to the terms of this study protocol.

12th January 2009

Maria Åberg, MSc., PhL (Med. Lic.)
Clinical Study Team Leader
Astra Tech AB
Aminogatan 1, SE-431 21 Mölndal, Sweden
Tel.: +46 31 776 3345

Date
(Day Month Year)

12th January 2009

Anna-Karin Sundin, R.N
Therapeutic Area Manager Health Care
Astra Tech AB
Aminogatan 1, SE-431 21 Mölndal, Sweden
Tel.:+46 31 63292

Date
(Day Month Year)

12th January 2009

Gunilla Almqvist, MSc.
Director, Clinical Research & Regulatory Affairs
Astra Tech AB
Aminogatan 1, SE-431 21 Mölndal, Sweden
Tel.: +46 31 63306

Date
(Day Month Year)

12-01-2009

Professor Magnus Jacobsson, MD, PhD
Vice President Research & Development/ Medical Director
Astra Tech AB
Aminogatan 1, SE-431 21 Mölndal, Sweden
Tel.: +46 31 63197

Date
(Day Month Year)

This document contains confidential information, which should not be copied, referred to, released or published without written approval from Astra Tech AB. Investigators are cautioned that the information in this protocol may be subject to change and revision.

ASTRA
ASTRA TECH

Signature of International Coordinating Investigator and Principal Investigator

A prospective, randomized, controlled trial of retransfusion of intra-operatively collected filtered whole blood in total hip surgery

I agree to the terms of this study protocol. I will conduct the study according to the procedures specified herein, and according to the principles of the Declaration of Helsinki, ISO 14155 and local regulations.

Centre No.: 1

Signature:

12/01/2009

Date
(Day Month Year)

Dr. Rudolf .W. Poolman, MD, PhD
Consultant Orthopaedic Surgeon
Department of Orthopaedic Surgery
Onze Lieve Vrouwe Gasthuis (OLVG)
1e Oosterparkstraat 279/1091 HA Amsterdam
Postbus 95500/1090 HM Amsterdam
Netherlands
Tel.: +31205999111

This document contains confidential information, which should not be copied, referred to, released or published without written approval from Astra Tech AB. Investigators are cautioned that the information in this protocol may be subject to change and revision.

ASTRA
ASTRA TECH

Signature of Principal Investigator

A prospective, randomized, controlled trial of retransfusion of intra-operatively collected filtered whole blood in total hip surgery

I agree to the terms of this study protocol. I will conduct the study according to the procedures specified herein, and according to the principles of the Declaration of Helsinki, ISO 14155 and local regulations.

Centre No.: 2

Signature:

Drs. Bregje J.W. Thomassen, MSc
Scientist, Afd. Orthopedie,
MC Haaglanden
P.O.Box 432
2501 CK DEN HAAG
Netherlands
Tel.: +31703303109

19 Jun 2009
Date
(Day Month Year)

This document contains confidential information, which should not be copied, referred to, released or published without written approval from Astra Tech AB. Investigators are cautioned that the information in this protocol may be subject to change and revision.

ASTRA
ASTRA TECH

Signature of Principal Investigator

A prospective, randomized, controlled trial of retransfusion of intra-operatively collected filtered whole blood in total hip surgery

I agree to the terms of this study protocol. I will conduct the study according to the procedures specified herein, and according to the principles of the Declaration of Helsinki, ISO 14155 and local regulations.

Centre No.: 3

Signature:

12-Jan-09
Date
(Day Month Year)

Dr. Peter Pilot, PhD
Senior scientist, afd. Orthopedie,
Reinier de Graaf Gasthuis (RdGG)
Pb 5501, 2600 GA Delft
Netherlands
Tel.: +31152603257

This document contains confidential information, which should not be copied, referred to, released or published without written approval from Astra Tech AB. Investigators are cautioned that the information in this protocol may be subject to change and revision.

ASTRA
ASTRA TECH

Signature of Principal Investigator

A prospective, randomized, controlled trial of retransfusion of intra-operatively collected filtered whole blood in total hip surgery

I agree to the terms of this study protocol. I will conduct the study according to the procedures specified herein, and according to the principles of the Declaration of Helsinki, ISO 14155 and local regulations.

Centre No.: 4

Signature:

Dra. Elvira Bisbe, MD
Consultant of Anesthesiology and Reanimation Department
Servicio de Anestesiología
Hospital Universitario Mar- Esperança (IMAS)
Passeig marítim 25, 08003 Barcelona
Spain
Tel.: +34 93 2483350

24-1-09
Date
(Day Month Year)

This document contains confidential information, which should not be copied, referred to, released or published without written approval from Astra Tech AB. Investigators are cautioned that the information in this protocol may be subject to change and revision.

ASTRA
ASTRA TECH

Signature of Principal Investigator

A prospective, randomized, controlled trial of retransfusion of intra-operatively collected filtered whole blood in total hip surgery

I agree to the terms of this study protocol. I will conduct the study according to the procedures specified herein, and according to the principles of the Declaration of Helsinki, ISO 14155 and local regulations.

Centre No.: 5

Signature: [signature] 26.01.2009

Dr. Ketil Holen, MD
St. Olavs Hospital
Ortopedisk avdelning
Olav Kyrres gate 17, 7006 Trondheim
Norway
Tel.: +4772574394

Date
(Day Month Year)

This document contains confidential information, which should not be copied, referred to, released or published without written approval from Astra Tech AB. Investigators are cautioned that the information in this protocol may be subject to change and revision.

ASTRA
ASTRA TECH

Signature of Principal Investigator

A prospective, randomized, controlled trial of retransfusion of intra-operatively collected filtered whole blood in total hip surgery

I agree to the terms of this study protocol. I will conduct the study according to the procedures specified herein, and according to the principles of the Declaration of Helsinki, ISO 14155 and local regulations.

Centre No.: 6

Signature:

13.1.09

Associate Professor Josef Grohs, MD
Department of Orthopaedic Surgery
Medical University Vienna
Währinger Gürtel 18-20
1090 Wien
Austria

Date
(Day Month Year)

This document contains confidential information, which should not be copied, referred to, released or published without written approval from Astra Tech AB. Investigators are cautioned that the information in this protocol may be subject to change and revision.

A company in the AstraZeneca Group

ASTRA
ASTRA TECH

CLINICAL STUDY PROTOCOL: APPENDIX B

Study Product:	Sangvia®
Study Code:	YA-DRA-0001
Version No.:	2
Version Date:	12th January 2009

Appendix B
Investigators and Study Administrative Structure

INVESTIGATORS AND STUDY SITES

**Centre No.**	**Centre Name and address**	**Name** (First name, Last name)	**Qualifications**	**Present position**	**Role in the study**
1	Department of Orthopaedic Surgery<br>Onze Lieve Vrouwe Gasthuis (OLVG)<br>1e Oosterparkstraat 279/1091 HA Amsterdam<br>Postbus 95500/1090 HM Amsterdam<br>Netherlands	Rudolf W. Poolman	MD, PhD	Consultant Orthopaedic Surgeon	International Coordinating Investigator/ Principal Investigator
		S.J. Ham	MD, PhD	Consultant Orthopaedic Surgeon	Investigator
		C.P. van der Hart	MD	Consultant Orthopaedic Surgeon	Investigator
		AE. B Kleipool	MD	Consultant Orthopaedic Surgeon	Investigator
		F.R. De Meulemseester	MD	Consultant Orthopaedic Surgeon	Investigator
		F. de Nies	MD	Consultant Orthopaedic Surgeon	Investigator
		W.J. Willems	MD, PhD	Consultant Orthopaedic Surgeon	Investigator
2	Afd. Orthopedie, MC Haaglanden	Bregje J.W. Thomassen	MSc	Scientist Orthopedic Department	Principal Investigator
	P.O.Box 432	S.B. Keizer	MD	Orthopaedic surgeon	Investigator
	2501 CK DEN HAAG	J.W.A. Swen	MD	Orthopeadic surgeon	Investigator
	Netherlands	P.H.C. den Hollander	MD	Orthopaedic surgeon	Investigator

ASTRA ASTRA TECH

Centre No.	Centre Name and address	Name (First name, Last name)	Qualifications	Present position	Role in the study
3	Reinier de Graaf Gasthuis (RdGG)<br>afd. Orthopedie<br>Pb 5501<br>2600 GA Delft<br>Netherlands	Peter Pilot	PhD	Senior scientist Orthopaedics department	Principal Investigator
		Rolf M. Bloem	MD, PhD	Orthopaedic surgeon	Investigator
		S.B.W. Vehmeijer	MD, PhD	Orthopaedic surgeon	Investigator
		R.L. te Slaa	MD, PhD	Orthopaedic surgeon	Investigator
		R.L.M. Deijkers	MD, PhD	Orthopaedic surgeon	Investigator
		H. Verburg	MD	Orthopaedic surgeon	Investigator
		J.A. Niesten	MD	Orthopaedic surgeon	Investigator
		S.J. Peters	MD	Resident orthopaedic surgeon	Investigator
		J. Breevaart v/d Bravenboer	MD	Resident orthopaedic surgeon	Investigator
		J. Wolkenfelt		Resident orthopaedic surgeon	Investigator
		M.C.M van Kessel	MSc	Junior scientist	Investigator
4	Servicio de Anestesiología<br>Hospital Universitario Mar-Esperança (IMAS)<br>Passeig marítim, 25<br>08003 Barcelona. Spain	Elvira Bisbe	MD	Consultant of Anaesthesiology and Reanimation Department	Principal Investigator
		Pau Rigol	MD	Trauma and orthopaedic resident	Investigator
		Carlos Mestre	MD	Orthopaedic Surgeon Chief	Investigator
		Marc Sadurni	MD	Consultant of Anaesthesiology	Investigator
		Jordi Moreno	RN	Supervisor	Study Nurse
		Caridad Vidal	RN	Orthopaedic surgery nurse	Study Nurse
		Rosa Villanueva	RN	Orthopaedic surgery nurse	Study Nurse

Centre No.	Centre Name and address	Name (First name, Last name)	Qualifications	Present position	Role in the study
5	St. Olavs Hospital<br>Ortopedisk avdelning<br>Olav Kyrres gate 17<br>7006 Trondheim<br>Norway	Ketil Holen	MD, PhD	Consultant Orthopaedic Surgeon	Principal Investigator
		Ivar Hanssen	MD	Consultant Orthopaedic Surgeon	Investigator
		Kaj Johanssen	MD	Consultant Anaesthesiologist	Investigator
		Britt Fyhn	RN	Anestsisykepleiere	Study Nurse
		Lise Høvik	RN	Anestsisykepleiere	Study Nurse
6	Department of Orthopaedic Surgery<br>Medical University Vienna<br>Währinger Gürtel 18-20<br>1090 Wien<br>Austria	AO Professor Josef Grohs	MD	Associate Professor, Department of Orthopaedic Surgery	Principal Investigator
		Dr. Martin Pfeiffer	MD	UK Orthopädie	Investigator
		Dr. Manuel Sabeti	MD	UK Orthopädie	Investigator
		Dr. Erdal Cetin	MD	UK Orthopädie	Investigator

OTHER INSTITUTIONS AND ESTABLISHMENTS

**Institution/Organization name**	**Address**	**Role in study**
StatCons; Mikael Åström	StatCons<br>c/o Mikael Åström<br>Högerudsgatan 8B<br>SE-216 18 Malmö<br>Sweden	Consultant Statistician
Premier Research	Premier Research Group plc / D-TARGET SA<br>En Chamard 55C<br>1442 Montagny-près-Yverdon<br>Switzerland	Site selection, Centre No. 6
